# Spontaneous Up states *in vitro*: a single-metric index of the functional maturation and regional differentiation of the cerebral cortex

**DOI:** 10.3389/fncir.2015.00059

**Published:** 2015-10-13

**Authors:** Pavlos Rigas, Dimitrios A. Adamos, Charalambos Sigalas, Panagiotis Tsakanikas, Nikolaos A. Laskaris, Irini Skaliora

**Affiliations:** ^1^Neurophysiology Laboratory, Center for Basic Research, Biomedical Research Foundation of the Academy of AthensAthens, Greece; ^2^Neuroinformatics Group, Aristotle University of ThessalonikiThessaloniki, Greece; ^3^School of Music Studies, Aristotle University of ThessalonikiThessaloniki, Greece; ^4^AIIA Lab, Department of Informatics, Aristotle University of ThessalonikiThessaloniki, Greece

**Keywords:** development, adolescence, aging, persistent activity, spontaneous network activity, cerebral cortex, oscillations

## Abstract

Understanding the development and differentiation of the neocortex remains a central focus of neuroscience. While previous studies have examined isolated aspects of cellular and synaptic organization, an integrated functional index of the cortical microcircuit is still lacking. Here we aimed to provide such an index, in the form of spontaneously recurring periods of persistent network activity -or Up states- recorded in mouse cortical slices. These coordinated network dynamics emerge through the orchestrated regulation of multiple cellular and synaptic elements and represent the default activity of the cortical microcircuit. To explore whether spontaneous Up states can capture developmental changes in intracortical networks we obtained local field potential recordings throughout the mouse lifespan. Two independent and complementary methodologies revealed that Up state activity is systematically modified by age, with the largest changes occurring during early development and adolescence. To explore possible regional heterogeneities we also compared the development of Up states in two distinct cortical areas and show that primary somatosensory cortex develops at a faster pace than primary motor cortex. Our findings suggest that *in vitro* Up states can serve as a functional index of cortical development and differentiation and can provide a baseline for comparing experimental and/or genetic mouse models.

## Introduction

Spontaneous network activity during quiescent states of the brain is a ubiquitous characteristic of both developing and mature cortical networks (Steriade et al., 1993b; Yuste et al., 2005; Egorov and Draguhn, 2013). It exhibits reproducible spatiotemporal patterns such as oscillations in various frequency bands (Steriade, 2006), synchronous discharges (Sanchez-Vives and McCormick, 2000), or waves propagating both within and across columns and cortical areas (Lampl et al., 1999; Massimini et al., 2004). Such spontaneous cortical activity is supported by recurrent circuits and seems to have a dual functional role depending on the maturational stage of the brain. During early development, when neuronal circuits are being established, the spatiotemporal structure of this activity and the resulting patterned alterations of intracellular calcium concentration are thought to regulate neuronal survival, growth, and synapse formation, and thus neural connectivity (Ben-Ari, 2002; Owens and Kriegstein, 2002; Cang et al., 2005; Feller and Scanziani, 2005; Demas et al., 2006; Nicol et al., 2007; De Roo et al., 2009; Yang et al., 2009; Ackman et al., 2012). In the mature brain, this activity has an active role in information processing: through its effect on neuronal conductances and membrane potential it provides a mechanism for modification of neuronal excitability (Shu et al., 2003a; Haider et al., 2006; Rigas and Castro-Alamancos, 2009). In this way it is thought to provide the neuronal context within which external signals are processed and interpreted (McCormick, 2005; Haider et al., 2007).

Among the many patterns of spontaneous cortical activity that have been reported, recurring Up/Down states seem unique in several respects: they are present throughout life, in all mammalian species tried (human (Amzica and Steriade, 2002; Cash et al., 2009; Moore et al., 2011, 2014), monkey (Kitano et al., 2002), cat (Steriade et al., 1993b), rat (Metherate and Ashe, 1993; Cunningham et al., 2006; Sheroziya et al., 2009), mouse (Rigas and Castro-Alamancos, 2007, 2009; Gibson et al., 2008; Watson et al., 2008; Ruiz-Mejias et al., 2011), ferret (Sanchez-Vives and McCormick, 2000; Haider et al., 2006), and in all cortical areas examined so far (Massimini et al., 2004; Ruiz-Mejias et al., 2011). In addition, spontaneous Up/Down states are routinely observed both *in vivo*, during quiescent states of the brain such as slow wave sleep (Steriade et al., 1993b, 2001), quiet wakefulness (Crochet and Petersen, 2006), or anesthesia (Metherate and Ashe, 1993; Haider et al., 2006), but also *in vitro*, in acute (Sanchez-Vives and McCormick, 2000; Cunningham et al., 2006; Rigas and Castro-Alamancos, 2007; Mann et al., 2009) and even organotypic slices (Johnson and Buonomano, 2007; Gireesh and Plenz, 2008), indicating they are a robust phenomenon and that mechanisms are in place to actively maintain these states. Importantly, spontaneous Up/Down states are intrinsic to the cortex and occur naturally, as a result of the recurrent connectivity of the neuronal circuits and the membrane and synaptic properties of its constituent elements and as such it is thought to represent the default activity of cortical networks (Steriade et al., 1993b; Sanchez-Vives and McCormick, 2000; Yuste et al., 2005; Rigas and Castro-Alamancos, 2007; Mann et al., 2009; Favero and Castro-Alamancos, 2013).

In the present study we examined spontaneous Up state activity throughout the entire lifespan of the mouse, with the goal of generating a single-metric index of cortical functional maturation and aging. Our reasoning was that, since Up states are generated by local feedback excitation between pyramidal cells, controlled, and tuned by di-synaptic feedback inhibition intrinsic to the neocortex (Steriade et al., 1993b; Shu et al., 2003b; Sanchez-Vives et al., 2010), they could integrate and reflect the cellular, and synaptic changes that take place in local cortical circuits as the brain develops, matures, and ages. Furthermore, since ontogenetic changes in the individual elements of cortical circuits are well-characterized in mice, the resulting recurrent activity would be both predicted and explained by the developmental modifications in neuronal organization. To this end we recorded spontaneous Up states in acute slices from the first postnatal days to advanced ages (27 months). While previous studies have been performed under diverse experimental conditions, species, and ages, this is the first study that examines the region specific development of Up states in a single species and under identical experimental conditions.

## Materials and methods

### Animals

C57Bl/6J mice were bred in the animal facility of the Center for Experimental Surgery of the Biomedical Research Foundation of the Academy of Athens. The facility is registered as a breeding and experimental facility according to the Presidential Decree of the Greek Democracy 160/91, which harmonizes the Greek national legislation with the European Council Directive 86/609/EEC on the protection of animals used for experimental and other scientific purposes. The present study was approved by the Regional Veterinary Service, in accordance to the National legal framework for the protection of animals used for scientific purposes (reference number 2834/08-05-2013). Mice were weaned at 21 days old (do), housed in groups of 5–10, in 267 × 483 × 203 mm cages supplied with bedding material and kept at a 12–12 dark-light schedule. Food was provided *ad libitum*.

### Brain slice preparation

Coronal brain slices (400 μm) from primary somatosensory cortex of the whiskers (i.e., barrel cortex, S1BF; Anterior-Posterior from Bregma (A/P): 0.58–1.58 mm, Medial-Lateral (M/L): 2.5–4 mm) or primary motor cortex (M1; A/P: 1.54–0.74 mm, M/L: 1–2.75 mm) were prepared from male mice of ages ranging from the third postnatal day (3do) to 27 months (27mo). After the mouse was sacrificed (cervical dislocation for mice older than 10 days or decapitation for mice younger than 10 days), the brain was removed and placed in an oxygenated (95% O_2_–5% CO_2_) ice-cold dissection buffer containing, in mM: KCl 2.14; NaH_2_PO_4_.H_2_O 1.47; NaHCO_3_ 27; MgSO_4_ 2.2; D-Glucose10; Sucrose 200; and CaCl_2_.2H_2_O 2; osmolarity (mean ± sd): 298 ± 5 mOsm, pH: 7.4. Slices were cut using a vibratome (VT 1000S, Leica) and placed in a holding chamber with artificial cerebrospinal fluid (ACSF). The ACSF contained, in mM: NaCl 126; KCl 3.53; NaH_2_PO_4_.H2O 1.25; NaHCO_3_ 26; MgSO_4_ 1; D-Glucose 10 and CaCl_2_.2H2O 2 (osmolarity (mean ± sd): 317 ± 4 mOsm, pH: 7.4) and were left to recover at room temperature (RT: 24–26°C) for at least 1 h before use.

### *In vitro* electrophysiology

Following recovery, slices were transferred to a submerged chamber (Luigs and Neumann), where they were gravity-perfused at high flow rates (10–15 ml/min) to ensure optimal oxygenation of the cortical tissue (Hájos et al., 2009; Bregestovski and Bernard, 2012). Recordings were performed in “*in vivo* like” ACSF (composition as above but with 1 mM CaCl_2_), since this ionic buffer is thought to better mimic cerebrospinal fluid *in vivo* (Fishman, 1992; Somjen, 2004) and we and others have previously shown that under these conditions cortical slices are spontaneously active (Sanchez-Vives and McCormick, 2000; MacLean et al., 2005; Rigas and Castro-Alamancos, 2007; Mann et al., 2009; Fanselow and Connors, 2010; Sigalas et al., 2015). Recordings were performed at RT after at least 30 min of incubation in 1 mM [CaCl_2_] ACSF buffer. To stabilize slices we used a modified submerged type of chamber that included a surface of transparent silicone onto which up to four slices could be pinned. The advantage of this modification was that we could perform simultaneous recordings from different ages and/or brain regions thus maximizing the yield and permitting a direct comparison of the different experimental groups under identical conditions.

Spontaneous network activity was assessed by means of (i) local field potential (LFP) recordings (sampled at 10 kHz, band-passed filtered at 1 Hz–3 kHz) which were obtained from cortical layers II/III using low impedance (~0.5 MΩ) glass pipettes filled with ACSF; and (ii) visually guided whole-cell patch clamp recordings from layers II-IV cells, obtained using 7–10 MΩ impedance electrodes. Patch electrodes were filled with internal solution containing (in mM): 135 K-gluconate, 4 KCl, 2 NaCl, 0.2 EGTA, 5 Tris-phosphocreatine, 0.3 Tris-GTP, 10 HEPES, and 4 MgATP (290 mOsm). Signals were acquired and amplified (MultiClamp 700 B, Axon Instruments), digitized (Instrutech, ITC-18) and viewed on-line with appropriate software (Axograph). In some experiments, gabazine (1–10 μM) or CNQX (10 μM) was added to the ACSF to block GABA_*A*_ or AMPA receptor mediated activity, respectively. All reagents and drugs were purchased from Sigma except for KCl and K-gluconate, which were purchased from Carlo Erba Reagents and Fluka, respectively.

### Data analysis

**Figure 1:**
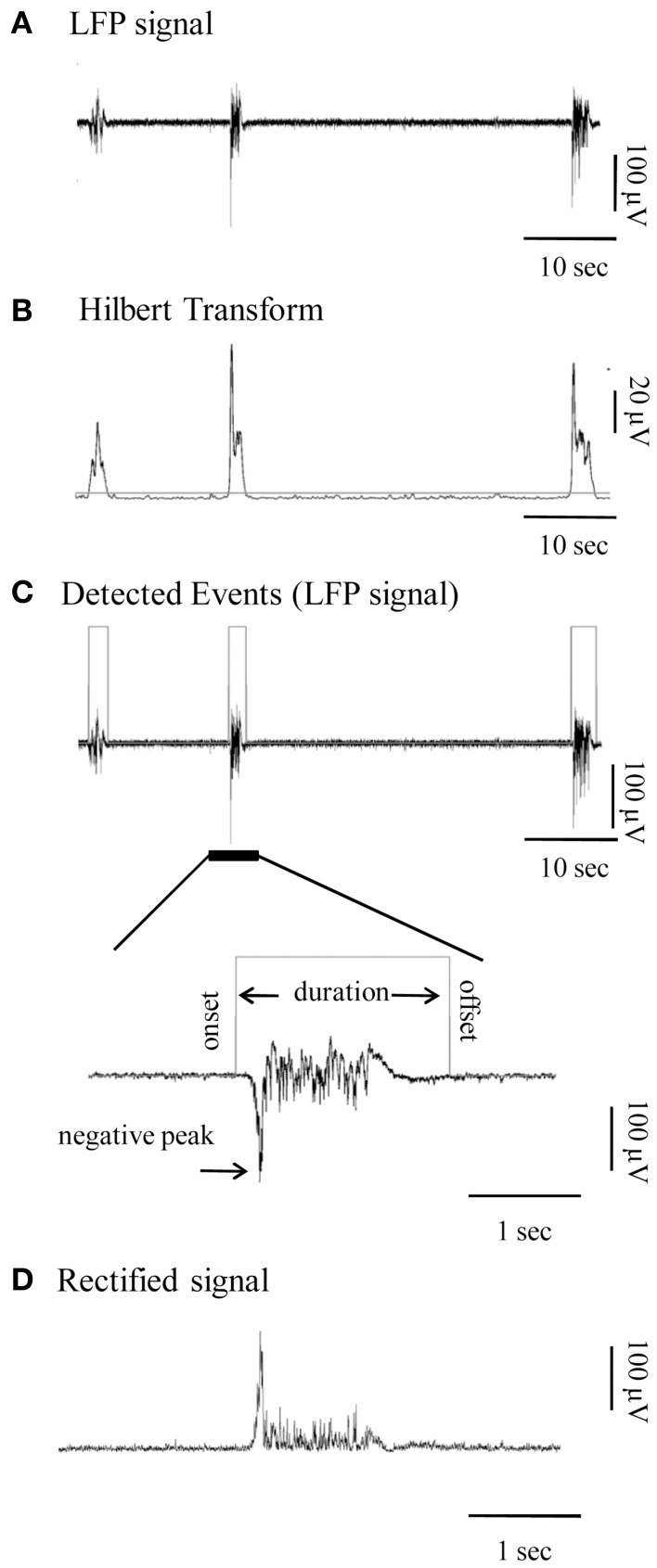
**Detection and quantification of the local field potential (LFP) events**. **(A)** Continuous LFP recording (1–200 Hz) of spontaneous bursts of activity from a cortical slice. **(B)** Hilbert Transform of the signal shown in **(A)** indicating the chosen threshold of detection (horizontal gray line). **(C)** Top panel: Automatically detected LFP events of the signal in **(A)** are outlined by gray rectangles. Bottom panel: Signal at high magnification provides view of individual Up state. Gray line is the automatically detected onset and offset of the event, on the basis of which duration is calculated. **(D)** Rectified signal (absolute valued signal) of the Up state, from which the rectified area is calculated.

For visualization and analysis of spontaneous events, traces were exported to MatLab format and analyzed with custom-made MatLab scripts that automatically detected the LFP events and marked their onsets and offsets. The data was first pre-processed by low-pass-filtering (at 200 Hz; third order Butterworth filter) and the DC offset subtracted (Figure 1A). Detection of individual LFP bursts was performed with the following automated method: firstly, the signal was transformed using the Hilbert Transform (Oppenheim et al., 1999) in order to estimate its envelope. This is a linear operation that takes a signal *u(t)* and transforms it to *H(u(t))*, in the same (time) domain. The Hilbert Transform has been successfully applied for latency analysis (van Drongelen et al., 2007; Recio-Spinoso et al., 2011) in neurophysiological signals and is one of the basic tools in Fourier analysis, providing a concrete means for realizing the harmonic conjugate of a given function or Fourier series. Secondly a threshold was applied so as to detect signal segments with fluctuation values larger than the 40% of the standard deviation of the entire signal (Figure 1B). This threshold was calculated for each trace (data driven threshold) in order to ensure that the detection procedure is adjusted to the corresponding signal-to-noise ratio of each recording and to the specific properties of each time-series (e.g., size and frequency of events).

#### Parametric (featured-based) analysis of up states

For each LFP event we measured several parameters, which were used for subsequent statistical analysis: (i) duration, based on the automatically detected onset and offset, (ii) maximal negative peak, (iii) rectified area and (iv) spectral power. Some of the measured parameters are depicted in Figures 1C,D. Event amplitude in the rest of the text refers to the amplitude of the max negative peak. In addition, several further parameters were calculated: (i) occurrence of spontaneous events (number of events divided by the duration of the recording session), (ii) fraction of time (%) in the Up state, calculated as the added duration of all Up states divided by the total recording time, (iii) an overall Up state activity index calculated as the product of occurrence ^*^ mean rectified area of Up states within each recording; and (iv) degree of variability in each parameter, quantified by calculating the coefficient of variation (CV) within each recording session. The power spectrum of each event, estimated on the basis of Fourier Transform coefficients, is presented both as continuous spectra, and in the conventionally described frequency bands: delta (1–4 Hz), theta (4–8 Hz), alpha (8–12 Hz), beta (12–30 Hz) and gamma (30–100 Hz) range, normalized to the total power of each event in the 1–100 Hz range. The normalization procedure allows a direct comparison of the % differences of power, since LFP events within or between recordings can differ significantly in both amplitude and duration and thus in absolute power value.

Statistical analyses were performed using SPSS (version 17) software. Sample size was defined based on the number of slices and data were tested for normality using the Shapiro-Wilk test. Normally distributed data (*p* > 0.05) were analyzed with parametric statistics (ANOVA or *t*-test); whereas data that deviated from normality (*p* < 0.05) were analyzed with either non-parametric statistics (Mann-Whitney) for non-paired comparisons of two groups; or non-parametric factorial analyses for multiple group comparisons after transforming data according to the rules of the Aligned Rank Transformation (Wobbrock et al., 2011) using the ARTool software (http://depts.washington.edu/aimgroup/proj/art/).

The comparison of Up state activity in primary somatosensory (S1BF) and motor (M1) cortex was performed using the Up state index measure. For both cortices the developmental profile had the form of an inverted U with a peak around which the changes were more pronounced. In order to describe and compare the timing of this developmental progression we used the DataFit Curve Fitting and Data Plotting Software available online by Oakdale Engineering (http://www.oakdaleengr.com) and fitted a peak function *y* = *a* ^*^ exp(−0.5 ^*^ (ln(*x*/*b*)/*c*)^2^) where *x* is age in postnatal days. We identified the peak as the time at which the first derivative of this function equals to zero, i.e., $y^{\prime }=\frac{-ae^{-\frac{{\left(\ln(x)-\ln(b)\right)}^{2}}{2c^{2}}}(\ln(x)-\ln(b))}{c^{2}x}=0\Rightarrow x=b$ (using the online available derivative calculator (http://www.derivative-calculator.net/). Finally, to evaluate if the developmental time course for the two cortices is significantly different we performed a two-sample *t*-test using the peak parameter *b* and its respective standard deviation, on the assumption that Up state index values are normally distributed within each age group.

#### Non-parametric (feature-less) analysis: up states as dynamical trajectories in a multidimensional phase-space

We applied a generic algorithmic framework, previously adopted for mining information from multi-trial datasets (Laskaris et al., 2004), multisite recordings (Laskaris et al., 2008) and spike-sorting (Adamos et al., 2010), in order to represent Up states as dynamical trajectories. The algorithmic framework employs data-learning techniques to capture and summarize the morphological variations in an ensemble of signals, and has been recently advanced by engaging methodological principles from the non-linear dynamics approach (Stam, 2005; Laskaris et al., 2013).

The analysis builds around the idea of comparing Up state events in pairwise fashion, by expressing their dis/similarities as differences/similarities between the corresponding trajectories formed within a common phase-space. The estimated dissimilarity scores (obtained by dissimilarity index *w*_*dist*_, as described in Supplementary Text, Section I) are then exploited for: (i) identifying the particular event that could serve as representative Up state for each recording; (ii) deriving a prototypical Up state event for each age group (used for visualization purposes); and (iii) detecting data structure related with developmental stages. Both representative and prototypical Up states are actual recorded events. Note that this analysis focuses on the internal structure of Up state events and does not take into account their rate of occurrence. Here, we outline the involved steps, as adapted to the context of the present study. A flow chart of the analysis and a more detailed description is included in the Supplementary Material (Figure S1).

##### Step 1: Transforming Up states into dynamical trajectories: analysis at the level of individual recordings

After band-pass filtering (1–45 Hz) by applying a third order Butterworth filter in zero-phase mode, signal segments of variable duration were extracted so as to contain the detected Up states. Each segment was transformed to a dynamical trajectory based on time-delay embedding (Stam, 2005). With that procedure, the segment *x(t)*, *t* = 1.2,…T was represented as a sequence of multidimensional vectors *X*_*t*_ = [*x*(*t*), *x*(*t* + 1),…, *x*(*t* + *d*_*e*_)], formed by successive signal values (*d*_*e*_ is the embedding dimension parameter that controls the dimensionality of the vectors). In this way, the emphasis was put on the dynamics reflected by each event without posing any assumptions about its morphological characteristics, hence enabling us to overcome problems related to the variable length of the segments.

##### Step 2: Identification of representative and prototypical Up states

The step of trajectory formation within a common phase-space was followed by the pairwise comparisons among all events. This was accomplished by means of a non-parametric multivariate statistical test, the Wald-Wolfowitz test, that measured the overlap between two vectorial distributions corresponding to any pair of trajectories (Figure S2, Supplementary Text). A dissimilarity matrix D_w_ was formed, with the entry D_w_(i,j) indicating the dissimilarity index w_dist_ (i,j) that corresponded to the statistical comparison between *i*th and *j*th events. Using the dominant-set algorithm (Adamos et al., 2012), we detected the subset of events forming the most cohesive group (in terms of dynamical trajectories), and estimated the centroid (i.e., the event whose trajectory was the most similar with the rest within the dominant-set) which served as the representative Up state event for each recording. This procedure was repeated for all recordings, leading to a set of 108 representative waveforms. Subsequently, the previous information-mining steps were applied separately to each age group (based on the previously derived representative events) in order to identify the prototypical Up state event for each developmental stage and hence facilitate visual comparisons.

##### Step 3: Estimating the co-variation between Up states and age

In order to test the hypothesis that Up states systematically vary with development, we used all 108 representative Upstate waveforms (dynamical trajectories) from the eight age-groups in order to correlate the variations in Up state morphology with age. First, we formed a (108 × 108) dissimilarity matrix ^animals^**D**_w_ by comparing, in pairwise fashion, the trajectories of all representative Up state events. Then, we estimated the distance-correlation between the age of the animals and a vectorial embedding reflecting inter-animal dissimilarities in terms of dynamical trajectories, based on a recent method for covariation assessment (Székely et al., 2007; Székely and Rizzo, 2009; Li et al., 2012)^1^. The embedding was derived by mapping the representative waveforms in a coordinate space of d_r_-dimensions, based on eigen-analysis of the ^animals^D_w_(i.e., multidimensional scaling (MDS) (Laskaris and Ioannides, 2002). Subsequently, using the obtained embedding, all dissimilarities among age groups were measured using the w-index. An (8 × 8) dissimilarity matrix ^Groups^**D**_w_ was then formed, which encapsulated the inter-group differences regarding dynamical trajectories. That matrix was fed to a hierarchical (single linkage) clustering algorithm, so as to organize the age-groups according to the morphology of Up state events. The resultant dendrogram (see **Figure 8**) is suggestive about the staging of related developmental changes.

##### Step 4: Deducing developmental stage from Up state trajectories

Finally, based on the five (5) developmental stages detected and confirmed as mutually exclusive (see results Developmental Progression of Spontaneous Cortical Up States), an efficient data-learning technique (the extreme learning machine, ELM (Huang et al., 2012, 2014) was applied to further verify the correspondence between Up state morphology and developmental stage. To this end, the ELM was trained (and tested using a cross-validation scheme) to learn the mapping from MDS coordinates to one of the five developmental stages (Supplementary Text, Section III). The overall methodology depends on two parameters, namely the time-delay embedding d_e_ and dimensionality of MDS-based embedding d_r_. These were set as 6 and 16, respectively, based on bootstrapping (Figure S3, Supplementary Text, Section II). Due to the large volume of processing power required, all the necessary computations were implemented in ~okeanos cloud computing service (http://okeanos.grnet.gr).

## Results

### Spontaneous up states in acute cortical slices

**Figure 2:**
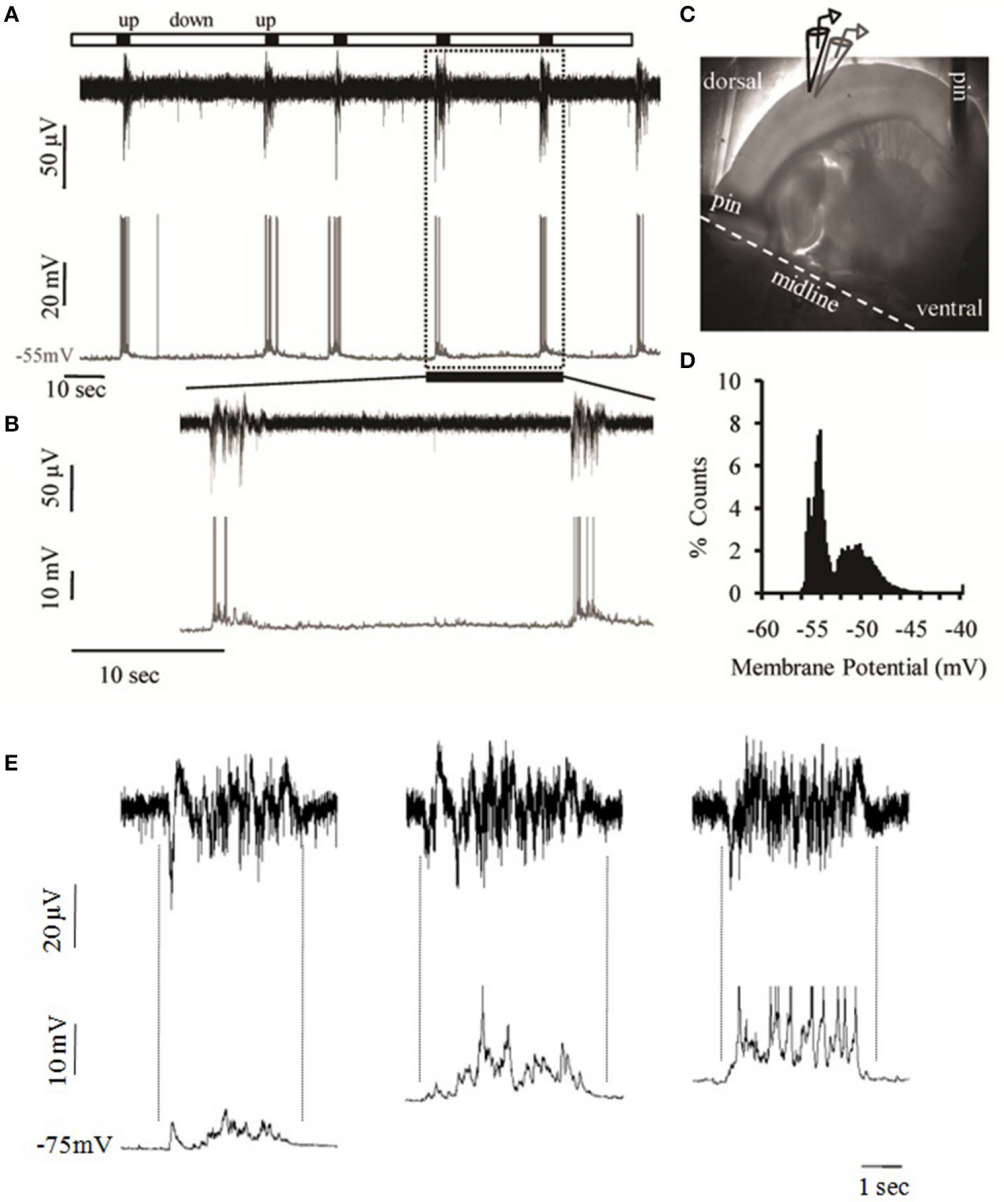
**Spontaneous cortical Up states ***in vitro*****. **(A)** Simultaneous extracellular (field potential, black) and intracellular (whole cell patch clamp, gray) recordings of spontaneous network activity from layers 2/3 and 4, respectively, of an S1BF cortical slice of a 15 days old male mouse. **(B)** Detail of recording period shown in **(A)** as defined by the dotted box in order to zoom into two individual Up states. **(C)** The mouse brain slice pinned onto a transparent layer of silicone and the arrangement of extracellular (black) and intracellular (gray) recording electrodes in cortical layers 2/3 and 4, respectively. **(D)** Example of the bimodal voltage distribution of a neuron's intracellular recording. The histogram represents the time spent at each observed membrane potential. The calculation is based on the distribution of the membrane potential of the cell recorded in (**A**, lower trace) as sampled from 300 ms segments of six Up states and six equally large segments of the baseline (Down state). **(E)** Spontaneous Up states recorded simultaneously extracellularly (upper traces); and intracellularly (lower traces) in successively more depolarized membrane potentials (left trace: −75 mV, middle trace: −65 mV, right trace: −60 mV). Dotted lines indicate the alignment between LFP and intracellular traces. The degree of alignment was quantified by calculating the correlation coefficients (CC) between the LFP and Vm recordings, which is a measure of the extent to which two measurement variables vary together. While CC values for either the Up or the Down states were extremely low (CC_Down_ = 0.03 ± 0.03, CC_Up_ = 0.02± 0.13), CC values for the transition phases were significantly higher (CC_Down−to−Up_ = −0.73±0.15, CC_Up−to−Down_ = 0.57± 0.22, *n* = 5), indicating that LFP and Vm values co-vary in near synchrony.

Local field potential (LFP) recordings from layer 2/3 of the mouse primary somatosensory cortex of the whiskers (i.e., barrel cortex, S1BF) revealed periodic bursts of persistent activity containing fast oscillations, or “Up states,” interspersed with quiescent periods, or “Down states” (Figure 2). Simultaneous whole cell recordings from cells in the same region confirmed the identity of such events as prolonged depolarizations of the neuronal membrane (Figures 2A,B). In the majority of cases these depolarized periods were accompanied by spike activity (*n* = 72.111, or 65%) with an average of 4.33 ± 3.35 action potentials per event (*n* = 72). The membrane potential followed the characteristic bimodal distribution typical of the Up/Down transition (Figure 2D) and the variance of Vm during the depolarized state was significantly larger (5.58 ± 3.18 mV) compared to the resting state (0.3 ± 0.18 mV), consistent with the literature (Metherate and Ashe, 1993; Lampl et al., 1999; Destexhe et al., 2003; Shu et al., 2003a).

Up states are synaptically mediated network events as indicated by the temporally constrained increase in synaptic activity, which is still present after hyperpolarization of the neuronal membrane and is also precisely aligned with the LFP signal (Figure 2E; Figure S4), in agreement with previous reports (Steriade et al., 1993b; Rigas and Castro-Alamancos, 2007). In addition, Up states are blocked by CNQX (10 μM, data not shown) and thus depend on AMPA-mediated transmission, in line with previous reports on Up states pharmacology (Steriade et al., 1993b; Sanchez-Vives and McCormick, 2000). Up state duration (ranging between 0.5 and 1.98 s) was comparable to *in vivo* recordings (Sanchez-Vives and McCormick, 2000; Timofeev et al., 2000). In contrast, Up state occurrence (ranging between 0.16 and 4 events/min) was significantly lower, but similar to recordings from isolated cortical slabs in living cats (Timofeev et al., 2000), consistent for preparations that contain a more restricted network.

Taken together these results indicate that acute cortical slices spontaneously produce alternating Up states of persistent network activity and Down states of generalized neural silence. In the rest of the paper we examine the hypothesis that this type of endogenous activity could provide a reliable signature of the functional maturation and differentiation of the cortical microcircuit.

### Developmental progression of spontaneous cortical up states

**Figure 3:**
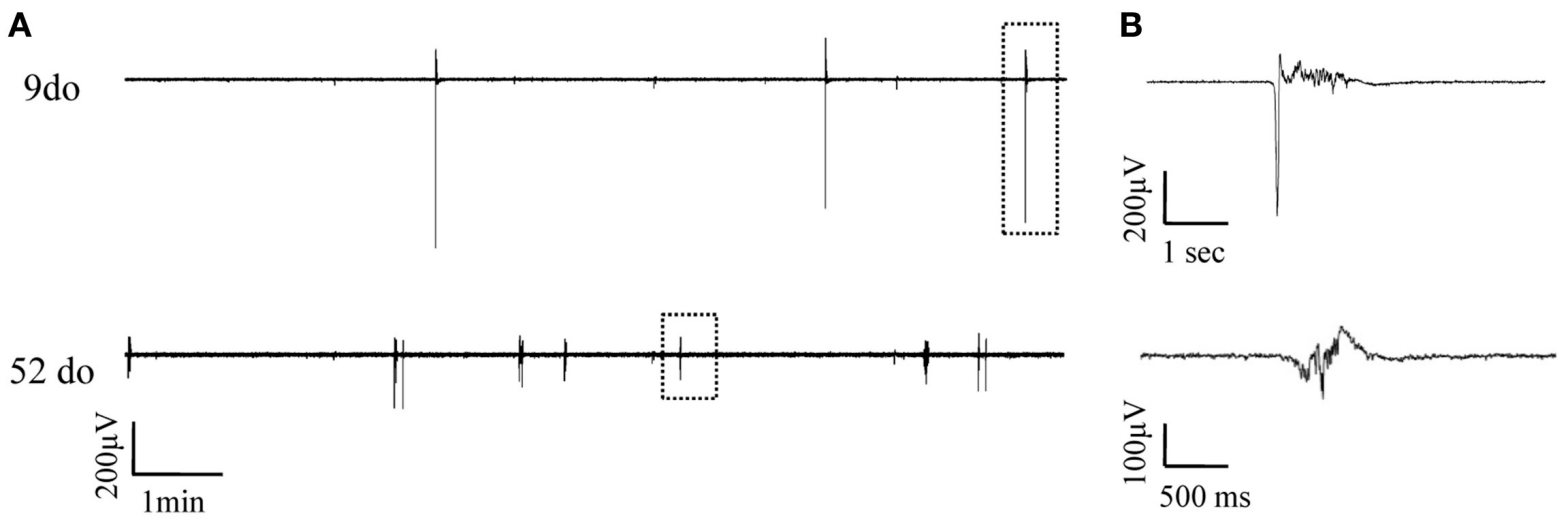
**Simultaneously recorded spontaneous Up states in slices from mice of two distinct ages. (A)** Continuous field potentials recorded from S1BF cortical slices of a 9do (top panel) and a 52do (middle panel) placed side-by-side in the recording chamber. **(B)** Higher magnification of individual Up states as designated by the dotted box in **(A)**.

**Figure 4:**
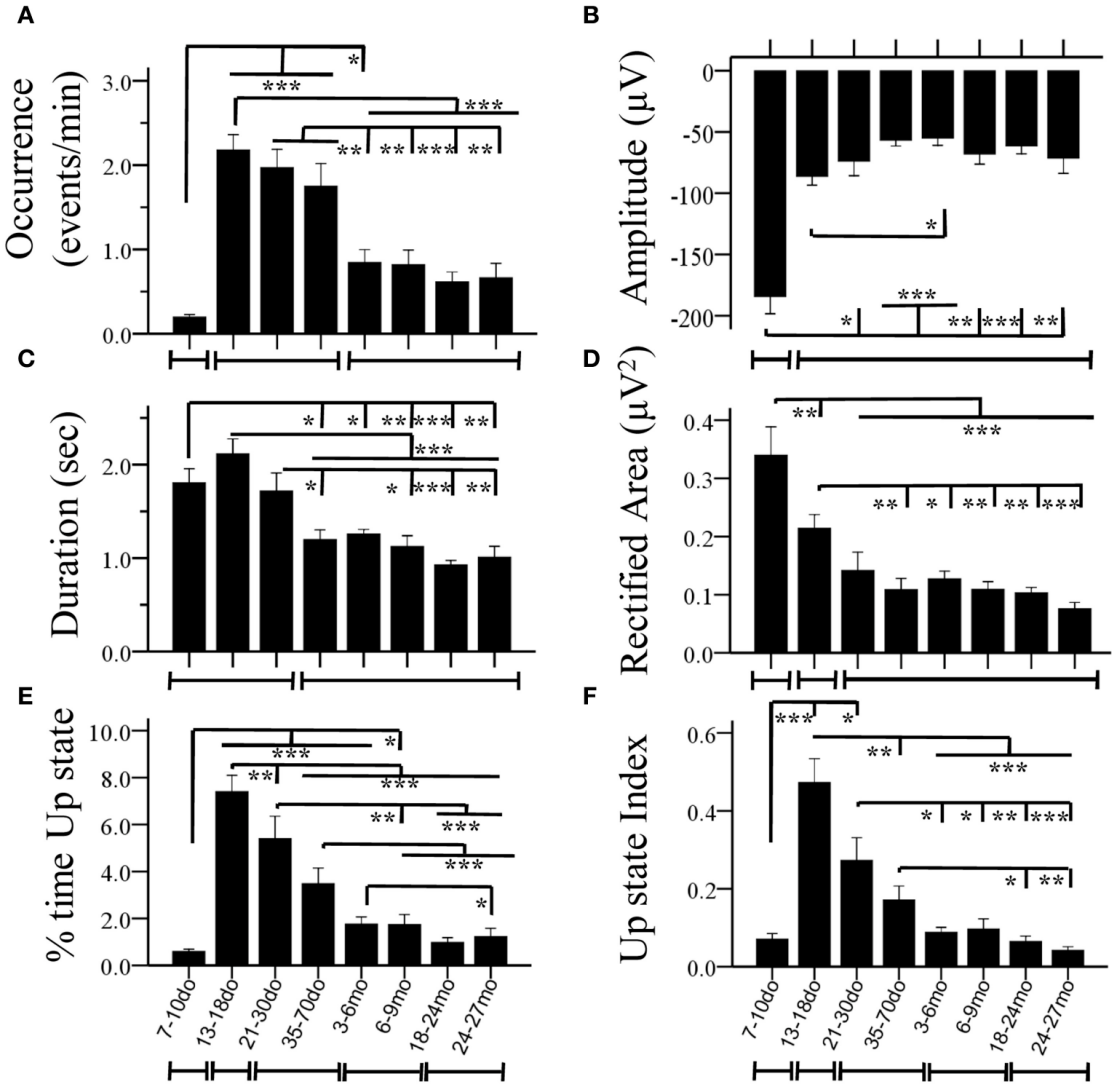
**Age-dependent changes of Up state parameters in the mouse S1BF cortex**. Bar plots illustrate mean values for occurrence **(A)**, amplitude **(B)**, duration **(C)**, rectified area **(D)**, percent (%) time spent in the Up state **(E)** and Up state index **(F)**, for the eight age groups. The lines below the X axis indicate the developmental periods with similar values (for each specific parameter) that are statistically different from preceding and following developmental stages (^*^p < 0.05, ^**^p < 0.01, ^***^p < 0.001).

**Figure 5:**
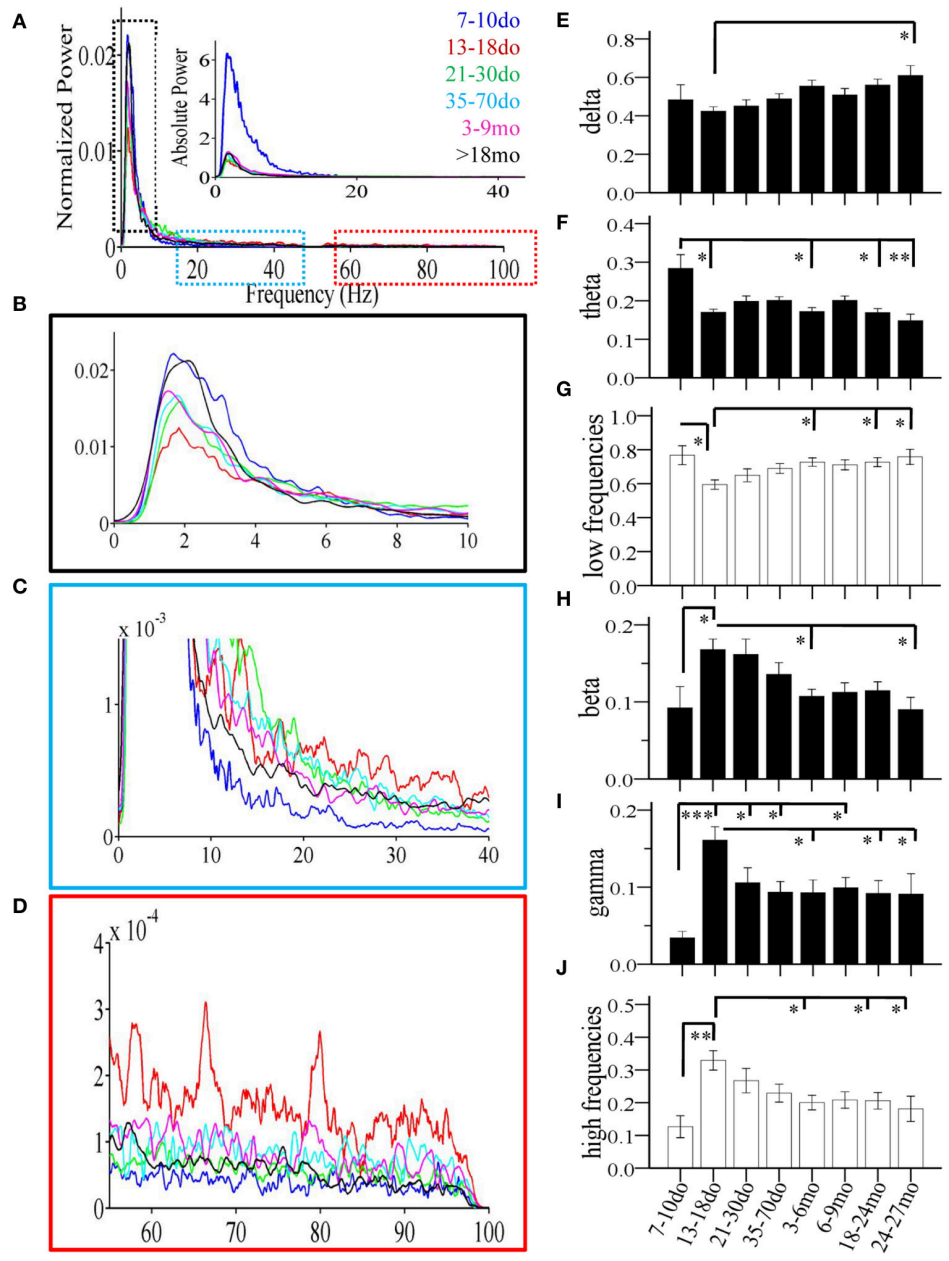
**Age-dependent changes of spectral power of individual Up states recorded in mouse S1BF cortex**. **(A)** Power spectrum (1–100 Hz) of Up states showing population data corresponding to the average fast-Fourier transform for each age group. Data are shown as normalized (i.e., normalized to total power) and absolute values (inset). Colors indicate the different ages. **(B–D)** Higher magnification of the relative power spectrum at ranges defined by the dotted boxes in **(A)**. **(E–J)** The developmental profiles of the mean normalized spectral power of Up states in the different frequency bands: delta, theta, beta, and gamma, or grouped in lower (delta + theta) and higher (beta + gamma) frequencies (^*^p < 0.05, ^**^p < 0.01, ^***^p < 0.001).

LFP recordings were obtained throughout the entire lifespan of the animal, from early postnatal development, through adolescence and into adult and old ages. Up state activity was absent in the first postnatal days (3–5do; *n*_slices_ = 6, *n*_animals_ = 3), and first appeared during the second week after birth. To enable statistical comparisons data were categorized into eight age groups, based on biologically relevant stages of development (Spear and Brake, 1983; Safranski et al., 1993; Spear, 2000; Adriani et al., 2004; Erzurumlu and Gaspar, 2012; Khan et al., 2013) from infancy to old age as follows: (i) 7–10 days old (do) (*n*slices = 8, *n*animals = 6): before the onset of sensory experience; (ii) 13–18do (*n*animals = 10, *n*slices = 14): centered around the onset of whisking and somatosensory experience; (iii) 21–30do (*n*_slices_ = 10, *n*_animals_ = 10): post-weaning, early puberty period; (iv) 35–70do (*n*_slices_ = 17, *n*_animals_ = 12): adolescence; (v) 3–6 months old (mo) (*n*_slices_ = 19, *n*_animals_ = 11): young adulthood; (vi) 6–9mo (*n*_slices_ = 13, *n*_animals_ = 7): maturity; (vii) 18–24mo (*n*_*slices*_ = 17, *n*_animals_ = 9): old age; and (viii) 24–27mo (*n*_slices_ = 10, *n*_animals_ = 7): advanced old age. Figure 3 shows an example of simultaneous field potential recordings from two cortical slices of different ages, while Figures 4, 5 illustrate the developmental progression of Up states' individual features.

#### Development of spontaneous cortical up states: parametric analysis

Statistical analysis revealed a significant main effect of age in practically all measures, including the occurrence of Up states [ANOVA, *F*_(7, 100)_ = 16.115, *p* < 0.001, Figure 4A], and their size, as reflected in measures of amplitude [ANOVA, *F*_(7, 100)_ = 5.911, *p* < 0.001, Figure 4B], duration [ANOVA, *F*_(7, 100)_ = 14.446, *p* < 0.001, Figure 4C], and rectified area [ANOVA, *F*_(7, 100)_ = 13.268, *p* < 0.001, Figure 4D]. These changes lead to significant alterations of the % of time that the local network is in an Up state [*x*^2(7)^ = 199.457, *p* < 0.001, Figure 4E), as well as the overall Up state index (occurrence ^*^ rectified area) [ANOVA, *F*_(7, 100)_ = 12.429, *p* < 0.001, Figure 4F]. Age also significantly modified the spectral content of cortical Up states, [delta: ANOVA, *F*_(7, 100)_ = 2.794, *p* < 0.05; theta: ANOVA, *F*_(7, 100)_ = 3.730, *p* < 0.01; beta: ANOVA, *F*_(7, 100)_ = 3.621, *p* < 0.01; gamma: ANOVA, *F*_(7, 100)_ = 4.634, *p* < 0.001; Figure 5]. The only parameter not affected by age was the normalized power in the alpha band [ANOVA, *F*_(7, 100)_ = 1.878, *p* > 0.05]. The changes in spectral content are also presented as combined lower (delta and theta) and higher (beta and gamma) frequency bands (Figures 5G,J) with significant effects on both, albeit in opposite directions [lower frequencies: ANOVA, *F*_(7, 100)_ = 2.985, *p* < 0.05; higher frequencies: ANOVA, *F*_(7, 129)_ = 3.731, *p* < 0.05]. These results indicate that individual parameters of Up state activity are significantly modified with age.

We next investigated the direction, magnitude and temporal progression of these modifications. In the following section we highlight the changes in Up state parameters at each age group compared to the preceding one. For ease of presentation the values of statistical comparisons are omitted from the main text and are indicated in table format in the Supplement Table 1. Unless explicitly stated, only statistically significant changes are described.

##### 7–10do

Spontaneous Up states first appeared during the second postnatal week as infrequent events of large amplitude and long duration (Figures 4A–C). These early Up states stood out by comparison to those of subsequent age groups in terms of larger size (Figures 4B,D, 5A inset) and lower proportion of high frequency oscillations (β/γ range; Figures 5H,I). In addition, they were much more stereotypic, as indicated by the lower variability in many parameters, including amplitude, duration, rectified area and θ band spectral power (Figure S5). In spite of their distinct appearance, these early Up states were abolished by CNQX and became more frequent after suppression of GABA_A_–mediated inhibition, like those at older ages (data not shown), suggesting they reflect a similar network phenomenon.

##### 13–18do

The period around the onset of sensory experience was characterized by dramatically increased levels of spontaneous activity. Up states manifested a 10-fold increase in occurrence (Figure 4A), which, despite the reduced amplitude and, consequently, rectified area (Figures 4B,D), resulted in a 10-fold increase in % time in Up state, and a six-fold increase in the Up state index (Figures 4E,F). Notably, this represents the maximum level of Up state activity throughout the lifespan. During this period, Up states also show significant alterations in spectral content, which shifted considerably in favor of higher (β/γ) frequencies (Figure 5).

##### 21–30do

Compared to the dramatic modifications of the previous period, the fourth postnatal week, highlighting the post-weaning early puberty period, was characterized by relative stability in spontaneous recurrent activity, as *post-hoc* analysis did not differentiate Up states from those of the previous period in any of the primary parameters (Figures 4A–D, 5; Supplement Table 1). Nevertheless, there were tendencies for further alterations toward a more mature phenotype in the power of the γ-band (Figure 5I), as well as in duration and rectified area, leading to significant reductions in % time in Up state (Figure 4E).

##### 35–70do

Modifications in Up state activity persisted through the period of adolescence with a significant (30%) reduction in Up state duration (Figure 4C). This likely contributed to the continuing decline in rectified area, % time in Up state and Up state index (which, however, do not reach statistical significance compared to the immediately preceding developmental stage; Figures 4D–F). There was also a tendency for reduced beta power content of individual events, toward adult levels (Figure 5H).

##### 3–6mo

Between the stages of adolescence and young adulthood Up states underwent further significant alterations in occurrence, which decreased by over 50% (Figure 4A), with a corresponding strong tendency for decrease in the % time in Up state activity and Up state index (Figures 4E,F; Supplement Table 1). There were also changes in the spectral power of Up states, with reduced proportion of high frequencies (Figure 5G) and correspondingly increased proportion of low frequencies (Figure 5J).

##### 6–9mo

The entire period of young adulthood and maturity was characterized by prolonged stability in Up state activity with no changes in any parameters (Figures 4, 5, Supplement Table 1).

##### 18–24mo

Following this sustained period of stability, overall levels of spontaneous activity exhibited a tendency to decline in aged animals. Average values for Up state occurrence and duration were about 20% lower (Figures 4A,C), although in the context of the entire lifespan the changes did not reach significance (Supplement Table 1). The spectral content of individual Up states in aged animals also remained unaltered compared to the adult levels (Figures 5E–J, Supplement Table 1).

##### 24–27mo

Up states in advanced old age were largely unchanged compared to the previous age group, except for the % time in Up state, which was significantly reduced compared to the young adult cortex (Figures 4, 5, Supplement Table 1).

**Figure 6:**
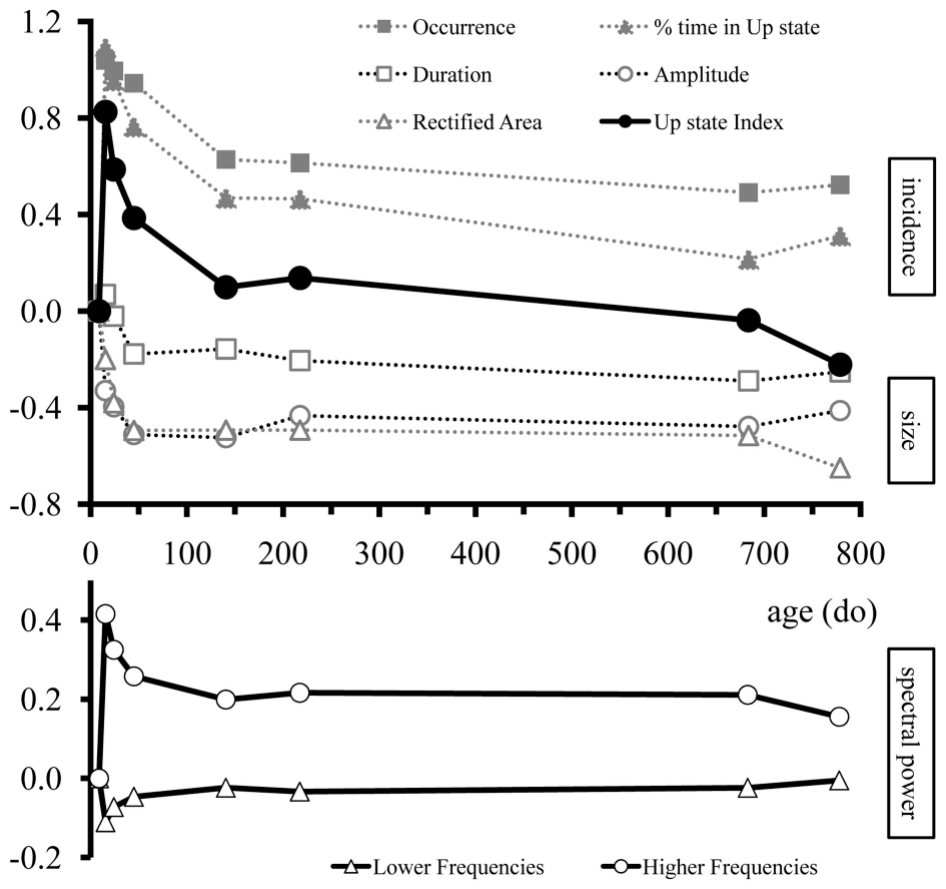
**Overview of age-dependent changes in Up state parameters of mouse S1BF cortex over the entire lifespan**. Upper panel: Developmental changes in occurrence, % time in Up state, duration, amplitude, rectified area, and Up state index. Lower panel: Developmental changes in the spectral content of Up states for the lower (1–8 Hz) and higher (13–100 Hz) frequencies. For illustration purposes values were normalized to the youngest age group (7–10do) and normalized values were log transformed so that traces would begin at zero. Positive and negative values indicate increase and decrease, respectively, compared to the age at which Up states first appeared. The different Up state parameters are grouped into three categories: incidence (occurrence and % time in Up state; solid gray symbols), size (amplitude, duration, rectified area; open gray symbols), and spectral content (lower and higher frequencies; bottom panel).

Taken together these results indicate that the specific features of cortical Up states are altered during development in a systematic way that, in combination, identify periods of intense change and periods of stability (Figure 6). It is noteworthy, that not all parameters were modified concurrently. For instance, the amplitude of Up states exhibited a drastic reduction between the second and third postnatal week and remained unaltered subsequently (Figures 4C, 6); while the occurrence of Up states exhibited a more complex developmental profile with a large increase after the second postnatal week, a plateau until the end of adolescence and a reduction to adult levels thereafter (Figures 4A, 6). Therefore, we next explored whether a qualitatively different, non-parametric analysis of Up state waveforms would reveal a consistent temporal profile.

##### Development of spontaneous cortical up states: non-parametric analysis

**Figure 7:**
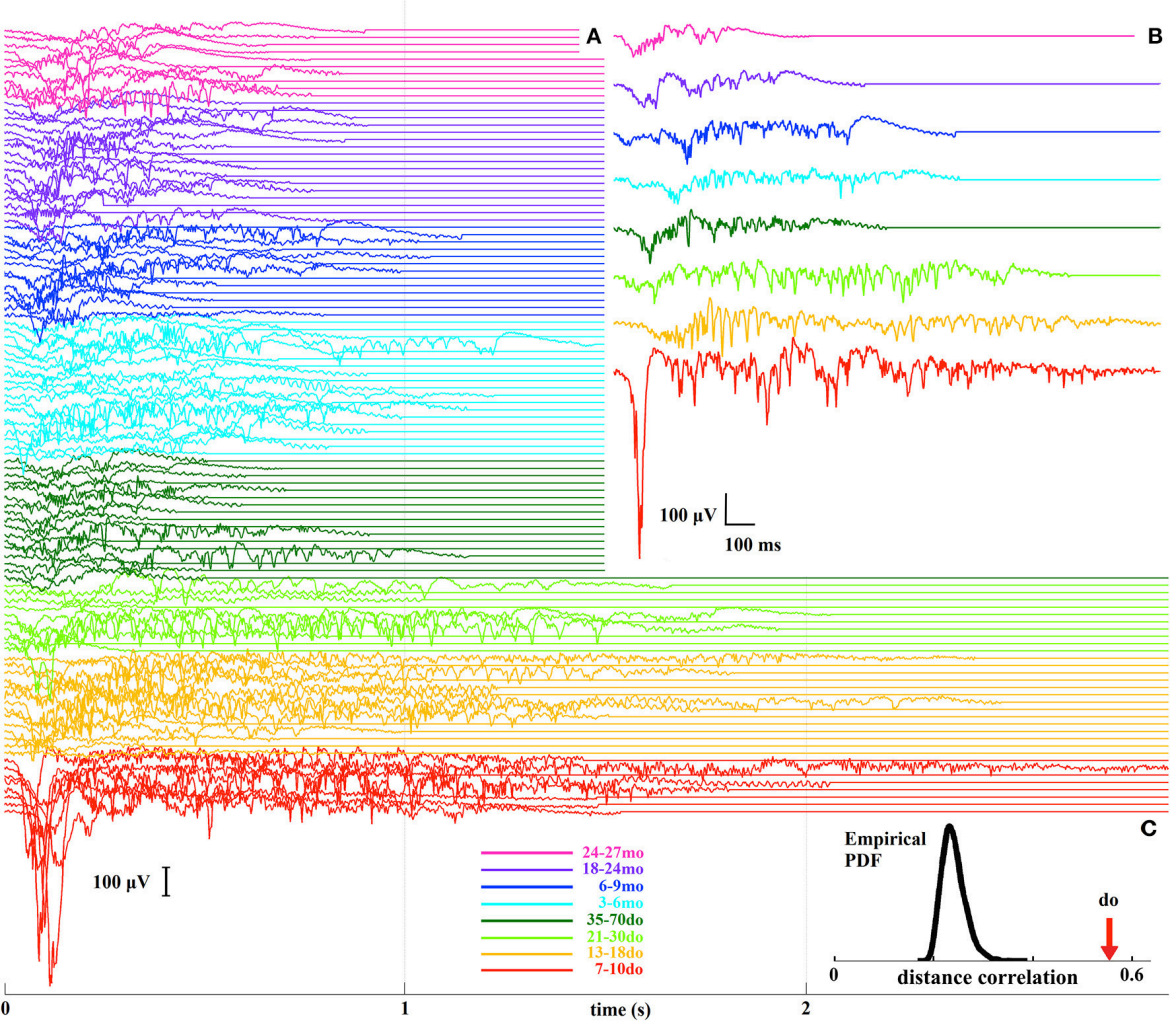
**(A)** A stack plot of all 108 representative Up states, where different colors indicate the different developmental stages. **(B)** Prototypical Up states for each of the eight age groups. **(C)** The bell-shaped curve illustrates the distribution (probability density function, PDF) of the distance-correlation values derived from the randomized assignment of Up state waveforms to age groups, and the arrow indicates the experimentally-derived distance-correlation index (do) for the original labeling.

In order to examine Up states as unified/whole entities we adopted a model-free information-mining procedure that does not rely on any assumptions about the nature of the signal, and which treats individual Up states as dynamical trajectories (Figure S2). A total of 108 LFP recordings from the eight age groups were analyzed leading to a set of 108 *representative Up states*, each being representative of all events in a given recording (Figure 7A). From these, a further set of eight *prototypical Up states*, each reflecting the “centroid” or “prototype” waveform of all events within a respective age-group, for visualization purposes (Figure 7B). Analysis of the mined waveforms revealed a covariation between age and Up state morphology, as estimated by means of distance-correlation index (d_o_ = 0.56; Figure 7C). Considering this is a non-linear correlation measure ranging from 0 to 1, the obtained value suggests that Up state waveforms depend strongly on age. To assess the statistical significance of the detected relationship, we repeated (10,000 times) the distance-correlation calculations after permuting the age-labels of the representative waveforms. The distribution of those 10,000 values in comparison to the actual distance-correlation index is shown in Figure 7C, indicating that the empirically derived d_o_ is highly statistically significant (*p* < 0.0001). This result provides clear evidence that development and maturation of the cortical microcircuit are reflected in spontaneous Up state dynamics.

**Figure 8:**
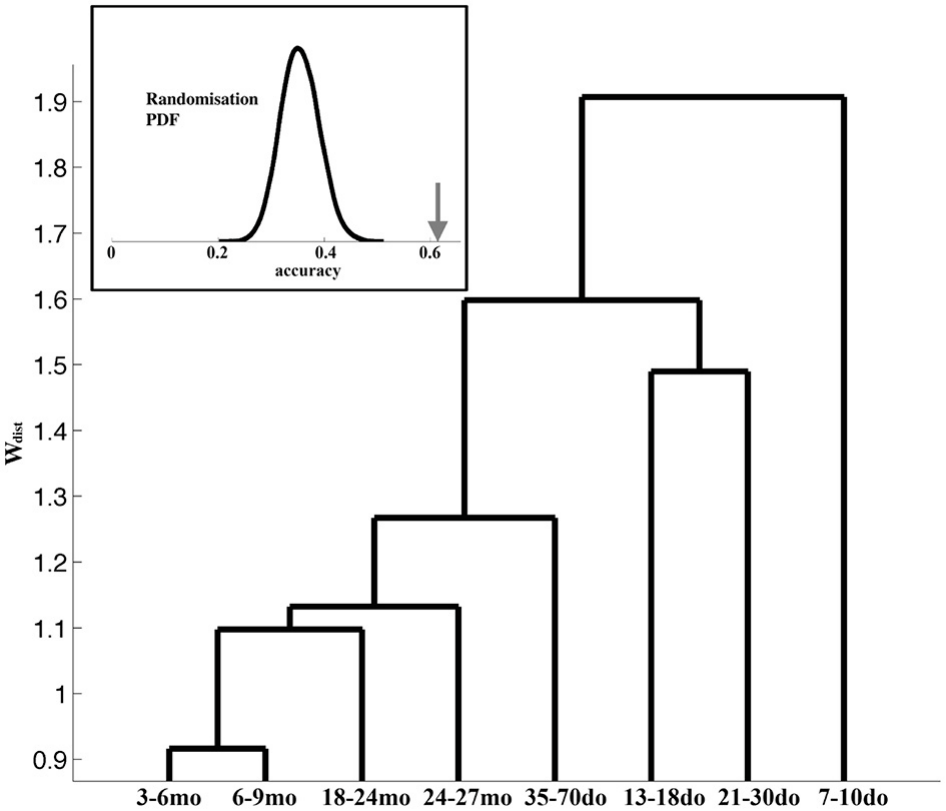
**Dendrogram reflecting the (dis)similarity relationships between the 8 age groups**. The furthest to the right a group is positioned, the stronger the indication for uniqueness. Inset: The bell-shaped curve illustrates the distribution (probability density function, PDF) of the accuracy scores reflecting the ability of the ELM classifier to learn the correspondence between the Up state waveforms and one of the five developmental stages after randomized labeling. In contrast, the arrow indicates the accuracy of the ELM classifier when trained with the originally labeled data.

We next asked the question whether the adopted grouping into the eight age categories is justified and/or optimal, by performing all inter-group comparisons of the corresponding representative Up states. The dendrogram in Figure 8 illustrates the hierarchy of inter-group dissimilarities as estimated from the dissimilarity index w_dist_. It can be observed that the lower-ranked dissimilarity scores have assembled in sequence the four oldest groups containing the adult and old animals, implying Up states from these groups are not sufficiently different and hence conform into a single entity. In contrast, the four youngest groups are assigned with higher-rank scores, suggesting they can be classified into distinct categories. This analysis indicates that Up state trajectories over the entire mouse lifespan can be assembled into five groups, corresponding to (i) the second postnatal week (7–10do), (ii) the third postnatal week (13–18do), (iii) the early puberty period (21–30do), (iv) adolescence (35–70do), and (v) maturity (3–27mo). Note that this analysis focuses on the dynamical trajectory of individual Up states as hence does not consider their rate of occurrence.

As a final step, in order to evaluate the robustness of the derived developmental profile of Up state dynamics, we attempted to train an ELM classifier to associate the Up state trajectory with the animal's developmental stage (as described in the Materials and Methods section). To assess the statistical significance of the outcome of the two-fold cross-validation scheme, we repeated the training of the same ELM classifier using random re-labeling. Figure 8 (inset) illustrates the contrast between the distribution of the accuracy values obtained for 10,000 permutations during which the representative waveforms were randomly assigned into the five developmental stages (bell-shape curve) and the experimentally observed accuracy (arrow). This result suggests that the ELM learned an association (between Up state trajectory and developmental stage) that reflects a true tendency inherent in the original data (*p* < 0.0001).

In summary, the results obtained from the two independent and complementary methods of analysis indicate that Up state activity is significantly and systematically modulated throughout the lifespan. The data mining analysis, which captures the overall structure of Up state waveforms, reveals that the most pronounced changes occur during the first developmental stages up until adolescence. The parametric analysis confirms this result and illustrates that the changes in dynamical trajectories are reflected in the amplitude, duration and spectral power of individual Up states. Beyond adolescence, Up state trajectories remain unchanged and the only further modification concerns their rate of occurrence which continues to decline during the transition to adulthood and again, but to a lesser extent, in old age. Hence, the combined outcome of the parametric analysis, as reflected in the two integrated measures of the fraction of active over silent periods and the Up state index, provides an accurate synthesis of the results and indicates that spontaneous Up state activity is altered during development, maturation and aging in a systematic way that identifies periods of intense change and periods of protracted stability of endogenous cortical dynamics.

#### Spontaneous up states in distinct cortical areas

In order to provide further evidence for the hypothesis that Up states could be used as a reliable signature of intracortical dynamics we examined this type of emergent activity in a second cortical region with distinct cytoarchitecture. Our reasoning followed the same logic; that since spontaneous Up states are generated by recurrent activation between excitatory neurons balanced by inhibition they should reflect the synaptic organization and network dynamics intrinsic to each cortical area. Therefore, we compared Up states in S1BF to those in the primary motor (M1) cortex, as these cortices are known to differ in a number of structural and functional parameters (Welker, 1971, 1976; Donoghue and Wise, 1982; Castro-Alamancos et al., 1995, 2007; Castro-Alamancos and Rigas, 2002; Kätzel et al., 2011; Herculano-Houzel et al., 2013).

We initially examined the two regions in adult (3–8 mo) mice (S1BF: *n* = 22 slices, *n* = 13 animals; M1: *n* = 18 slices, *n* = 14 animals) and found that M1 slices manifest a lower probability to exhibit spontaneous Up states [20 vs. 50%, *t*_(132)_ = 3.564, *p* < 0.01, Student's *t*-test]. In spontaneously active slices, M1 slices generate Up states of about 20% longer duration (1.25 ± 0.2 vs. 1.48 ± 0.43 s, *p* < 0.05, Student's *t*-test, Supplement Table 2), but nearly half the occurrence (0.87 ± 0.61 vs. 0.49 ± 0.32 (min^−1^), *p* < 0.05, Mann-Whitney), resulting in a lower % time in Up state (1.80 ± 1.23 vs. 1.24 ± 0.90%, *p* < 0.05, χ^2^ independence test) and a smaller Up state index (0.09 ± 0.05 vs. 0.05 ± 0.03, *p* < 0.05, Student's *t*-test, Supplement Table 2). In addition, Up states in M1 cortex show a power content moderately shifted toward higher frequencies compared to the S1BF (Supplement Table 2). These findings suggest that intrinsic differences in cortical networks are reflected in the emergent network dynamics in the form of spontaneous Up states.

**Figure 9:**
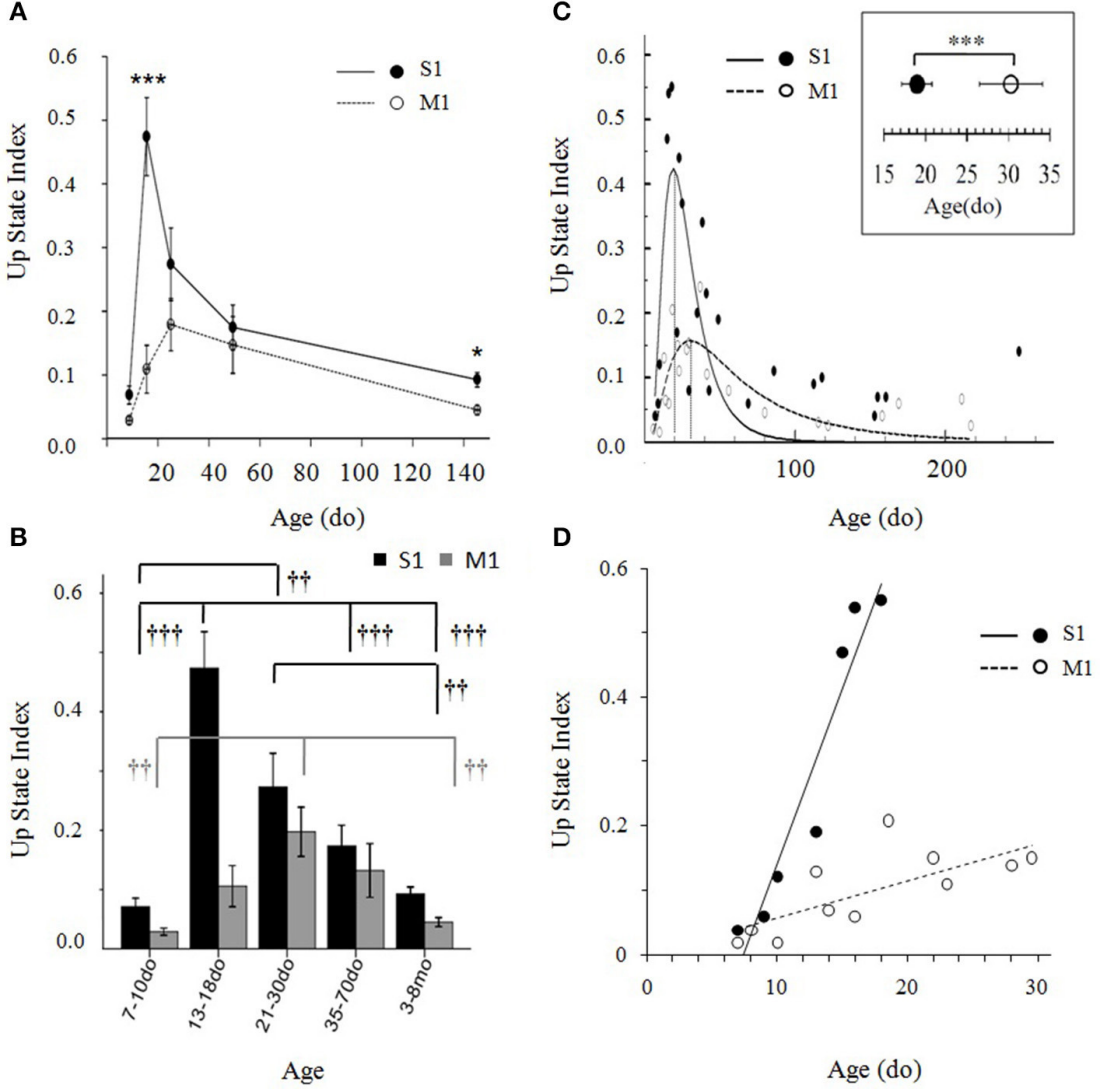
**(A)** Developmental trajectories of Up state index in primary somatosensory (S1BF) and primary motor (M1) cortex of the mouse during the first 5 months of life. Data are combined into five developmental groups (7–10do, 13–18do, 21–30do, 35–70do, 3–8mo) and plotted as the mean age of each group. *Post-hoc* pairwise comparison analysis of the significant age X cortex interaction differentiates the development of the two cortices during the third postnatal week (13–18do) (^***^p < 0.001) and adulthood (^*^p < 0.05). **(B)** Data in **(A)** depicted as bar plots. Symbols indicate the significance of the effect of age on Up state index within each cortex (levels of significance: †††*p* < 0.01, ††*p* < 0.01). **(C)** Up state index values of both cortices plotted as a continuum from early postnatal age until adulthood. Data points, each representing the mean Up state index at a given age, were fitted by peak functions (S1BF cortex: y = 0.42^*^ exp(−0.5^*^(ln(x/18.97)/0.53)^2^, *R*^2^ = 0.55, *p* < 0.001; M1 cortex: y = 0.16^*^exp(−0.5^*^(ln(x/30.3)/0.76)^2^, *R*^2^ = 0.60; *p* < 0.001). Inset: Statistical comparison the time of peak of Up state index in S1BF and M1 cortex: S1BF vs. M1 (mean ± sd): 19 ± 1.8do vs. 30 ± 7.4do. **(D)** Data plotted at higher temporal resolution for the ascending phase of the developmental trajectory of each cortex are described by linear regressions (S1BF: Y = 5.4^*^10^−2^ X −0.5, *R*^2^ = 0.91, *p* < 0.001; M1: Y = 5.9^*^10^−3^ X −1.8, *R*^2^ = 0.55, *p* < 0.01).

We then compared the Up state index in the two cortices from the second postnatal week until adulthood: 7–10do (S1: *n* = 8 slices, *n* = 6 animals; M1: *n* = 7 slices, *n* = 7 animals); 13–18do (S1: *n* = 14 slices, *n* = 8 animals; M1: *n* = 14 slices, *n* = 6 animals); 21–30do (S1: *n* = 10 slices, *n* = 9 animals; M1: *n* = 13 slices, *n* = 10 animals), 35–70do (S1: *n* = 17 slices, *n* = 12 animals; M1: *n* = 18 slices, *n* = 8 animals), and 3–8mo (S1: *n* = 22 slices, *n* = 13 animals; M1: *n* = 18 slices, *n* = 14 animals) (Figures 9A,B). A Two-Way ANOVA revealed a significant interaction between age and cortex type [*F*_(4, 126)_ = 7.885, *p* < 0.001] indicating that Up states in the two cortical regions follow different developmental trajectories (Figure 9A).

We next asked the question whether the differences are attributed to a temporal shift in time course and/or to a different rate of maturation. In both cortices, the developmental progression of Up state index has the form of an inverted U with an ascending and a descending phase (Figures 9A,B). A One-Way ANOVA performed separately for each region revealed a significant effect of age in both cortices, but a stronger impact of age on S1BF, as indicated by the higher *F*-value [S1BF cortex: *F*_(4, 66)_ = 15.638, *p* < 0.001; M1 cortex: *F*_(4, 60)_ = 6.459, *p* < 0.01, Figure 9B]. In addition, the modifications of Up state activity in the two regions had distinct temporal profiles: the increase in Up state index in S1BF reached statistical significance during the third postnatal week, whereas in M1 the increase became statistically significant later, during the fourth postnatal week (Figure 9B). These results suggest that changes in network function occur at a faster rate in S1 compared to M1. Hence, to further investigate the relative development in S1BF vs. M1 we performed an additional analysis at higher temporal resolution in order to estimate the precise time for the peak in Up state index for each cortical region (see Materials and Methods Section). When Up state index values were scatter plotted at a day-day resolution (Figure 9C), the data points were well-fitted by peak functions (**S1BF** cortex: y = 0.42^*^exp(−0.5^*^(ln(x/18.97)/0.53)^2^, *R*^2^ = 0.55, *p* < 0.001; M1 cortex: y = 0.16^*^exp(−0.5^*^(ln(x/30.3)/0.76)^2^, *R*^2^ = 0.60; *p* < 0.001). This allowed us to identify the peak of each trajectory at 19 ± 1.8 do for S1BF, and at 30 ± 7.4 do for M1 [*t*_(42)_ = −12.43, *p* < 0.001, Student's *t*-test]. Moreover, when we plotted Up state index values from the earliest ages tested until the respective peak for each region we found that the data were well-fitted by linear regressions (S1BF: Y = 5.4^*^10^−2^ X -0.5, *R*^2^ = 0.91, *p* < 0.001; M1: Y = 5.9^*^10^−3^ X −1.8, *R*^2^ = 0.55, *p* < 0.01) whose slopes showed a significant interaction between age and type of cortex [*F*_(2, 15)_ = 25.25, *p* < 0.001, ANCOVA). Therefore, this analysis confirmed that compared to M1, Up states in S1BF undergo both faster and stronger developmental changes during the early postnatal period (Figure 9D). Taken together, these results suggest that spontaneous Up state activity can differentiate the functional development of discrete cortical regions.

## Discussion

In the present study we used spontaneous Up state activity as a metric which captures functional differences in cortical microcircuits and examined, for the first time, the emergence and subsequent maturation of Up states in two distinct types of cortex. We find that endogenous Up state activity is under tight temporal and spatial regulation: it is systematically modified during development, suggesting ongoing modifications in cortical circuits past the stage of adolescence; and it also exhibits regional specificity, supporting the view that different cortical areas have distinct intrinsic organization and developmental trajectories. Hence, this work introduces a novel marker of network dynamics that should be useful as a baseline against which to compare cortical emerging activity in mouse models of neurodevelopmental and/or age-related disorders.

### Up states *in vivo* and in slices: technical considerations

Our aim in this study was to use an *in-vitro* model of endogenous activity, as this offers the possibility to examine distinct cortical areas in isolation from each other and from the thalamocortical loop, which plays a crucial role in the regulation of cortical states (Crunelli and Hughes, 2010). In addition, an *in-vitro* approach is more amenable to future pharmacological investigations of the underlying mechanisms, and also avoids the confounding effects of type and level of anesthesia, both of which are known to severely affect cortical oscillatory activity, and are rarely identical between animals of different ages (Steriade et al., 1993b; Friedberg et al., 1999; Greenberg et al., 2008; Gargiulo et al., 2012) The option of studying Up states *in-vitro* is justified since numerous publications from several labs including our own have revealed that cortical slices maintained in ACSF-like buffer spontaneously generate Up states that are very similar to those observed *in vivo*, during quiescent brain states or anesthesia (Sanchez-Vives and McCormick, 2000; Crochet and Petersen, 2006; Rigas and Castro-Alamancos, 2007; Mann et al., 2009; Ruiz-Mejias et al., 2011; Sigalas et al., 2015). The present study confirms the presence of this network activity and further demonstrates that it is possible to record spontaneous Up states in cortical slices from the entire mouse lifespan under identical conditions.

A potential shortcoming of slice recordings is an inadequate level of oxygenation of the cortical tissue (Ivanov and Zilberter, 2011). To avoid this problem we performed all experiments in high flow rates (10–15 ml/min). Previous studies have shown that perfusion rates correlate with local oxygen saturation and that at such flow rates the oxygen tension reaches a plateau, thus providing optimal conditions for neuronal responses and spontaneous network activity (Hájos et al., 2009; Ivanov and Zilberter, 2011). In addition, Up states in our study are highly similar in appearance, properties and pharmacological profile to that reported in many *in vitro* studies of Up states (Shu et al., 2003a; MacLean et al., 2005; Cunningham et al., 2006; Rigas and Castro-Alamancos, 2007, 2009; Compte et al., 2008; Mann et al., 2009; Fanselow and Connors, 2010); and also comparable to Up states recorded in anesthetized animals (Timofeev et al., 2000; Ruiz-Mejias et al., 2011). Both *in vivo* and *in vitro*, Up/Down states are characterized by a distinctive bimodal Vm distribution; they have an average duration between 1 and 6 s and they are state-dependent as they disappear during activated states of the brain, or after application of cholinergic agonists (Metherate and Ashe, 1993; Steriade et al., 1993a; Favero et al., 2012; Sigalas et al., 2015). The main difference between *in vivo* and *in vitro* studies is the frequency of recurring Up states which is significantly lower in the latter, but similar to recordings from isolated cortical slabs in living cats (Timofeev et al., 2000), consistent for reduced preparations that contain a more restricted network.

### Spontaneous up states as an emergent property of the developing cortical microcircuit

Our data revealed that spontaneous Up states are absent in cortical slices from newborn mice (3–5do) and first emerge during the second postnatal week. This finding appears to contradict previous reports of early cortical network activity, in both rodents and humans (Vanhatalo and Kaila, 2006; Sun and Luhmann, 2007). However, in all these studies cortical activity was observed under very different experimental conditions: early network oscillations (ENOs) with event kinetics similar to Up states were recorded during the first days after birth, but mainly in horizontal—rather than coronal—slices and under conditions of mild hypoxia—in contrast to our conditions of high flow rates for optimal oxygenation (Crépel et al., 2007; Alléne et al., 2008). In other cases, early network acitivty was either recorded *in vivo* (Vanhatalo and Kaila, 2006), or necessitated much thicker cortical *in vitro* preparations (intact cortices, or slices of at least 500–1000 μm thickness) (Dupont et al., 2006; Sun and Luhmann, 2007; Moore et al., 2011, 2014). This is in agreement with studies showing a direct link between the size of the network and the cortex's propensity to generate spontaneous Up states (Timofeev et al., 2000). Finally, in preparations more similar to ours, the presence of activity required pharmacological induction or elevated potassium concentrations concentrations (Hanganu et al., 2009).

The lack of spontaneous LFP activity in slices obtained from very young animals could reflect the immaturity of cortical circuitry, including the low numbers and ongoing maturation/differentiation of neurons and/or synapses (Blue and Parnavelas, 1983; Rice et al., 1985; De Felipe et al., 1997; Lyck et al., 2007; Bandeira et al., 2009; Okaty et al., 2009; Goldberg et al., 2011; Pangratz-Fuehrer and Hestrin, 2011). In addition, layer 5 pyramidal neurons in newborn mice (2–4do) are under maximal tonic inhibition Sebe et al., 2010, possibly discouraging the generation of synchronized epochs of persistent activity. Although a direct comparison to other early patterns of spontaneous activity was beyond the scope of the present study, our observation that early (7–10do) LFP events have distinct properties, including a larger size, different spectral content and lower variability, raises the possibility that these immature Up states may be related to the giant depolarizing potentials, a transient form of coordinated activity that dominate the cortex during the second postnatal week.

Taken together, our data suggest that Up states recorded in cortical slices can be viewed as an emergent property of the isolated cortical microcircuit that appear during the second postnatal week and gradually acquire their mature phenotype. This interpretation concurs with a number of studies showing that spontaneous activity is absent in immature networks and emerges gradually as the networks develop (Shu et al., 2003b; Johnson and Buonomano, 2007; Rochefort et al., 2009; Sheroziya et al., 2009) and suggest that the immature cortex may be more dependent on long range connectivity to sustain persistent network activity.

### Up state activity as a signature of cortical network development

The two independent ways of analyzing the data offer complementary information on the maturation of endogenous Up states and together suggest that these undergo the strongest modifications in early development and up to, and including, the period of adolescence. This is evident both from the magnitude of changes in the individual and integrated parameters, as well as from the analysis of Up state waveforms revealing the largest dissimilarity scores among the first four age groups. In contrast, the period of adulthood (3–9mo) is characterized by prolonged stability, with no changes in individual parameters and smallest dissimilarity scores. Adolescence seems to be a special case with some features similar to the post-weaning period (e.g., occurrence), others comparable to adulthood (e.g., duration) and others in between the two (e.g., Up state index, % time in Up state, beta power). The high-rank dissimilarity score further indicates that Up states during this period most likely constitute a distinct group. Consequently, our data suggest that cortical networks are under continuous rearrangement that extends well past the early postnatal stages and the traditionally defined critical periods (Hensch, 2004), through adolescence into the fully adult stage. Notably, this is in line with electroencephalographic (EEG) recordings in both mice and humans showing a decline in slow wave activity during adolescence (Feinberg and Campbell, 2010; Buchmann et al., 2011; de Vivo et al., 2014). This inferred re-organization of intracortical circuits during adolescence may account for the heightened vulnerability of this developmental stage to the emergence of a number of psychiatric disorders (Paus et al., 2008; Uhlhaas and Singer, 2012). It also suggests that cortical circuits might still be malleable enough during adolescence to allow interventions that would target neurodevelopmental defects.

At the end of the age spectrum, Up states in the oldest age groups (18–24 and 25–27mo) appear largely indistinguishable from those in adult animals both in terms of individual features and of dynamical trajectories. The decline in duration and occurrence reaches significance only when combined into the integrated parameters of % time in Up state or Up state index. Although, this seems to contradict our recent report describing a modest but significant decrease in duration in old animals (Sigalas et al., 2015), the discrepancy can be explained in terms of the different context of the statistical comparison (2 vs. 8 groups). Taken together these findings indicate that spontaneous Up state activity in cortical slices is a sensitive functional marker that may reflect periods of intense reorganization in intracortical circuitry (early development to adolescence) and periods of stability (adulthood and old age). The period of adolescence (35–70do) stands out as a transition between the two, suggesting that intracortical circuits continue to re-organize and that mice can not be considered fully adult before the third postnatal month. This has obvious implications for developmental studies and could account, at least in part, for disparate conclusions reached in studies using animals of different ages (e.g., MacLean et al., 2005; Rigas and Castro-Alamancos, 2007, 2009).

These developmental changes are contingent on the assumption that cortical slices at all ages are well-supplied in oxygen and are not metabolically compromised. While we can ensure identical conditions in all experiments, it is known that neonatal rodents are more able to cope with anoxia than adults (Hansen, 1976; Cherubini et al., 1989; Jiang et al., 1991). Therefore, an alternative interpretation is that differences in Up state activity we observe with maturation are a reflection of the developmental/metabolic differences in the ability to cope with shortages in oxygen. However, a number of observations render this alternative highly unlikely: (i) Our recording conditions were designed to ensure maximal oxygenation; whereas all studies that have indicated a differential ability to cope with hypoxia/anoxia have employed much harsher paradigms that explicitly aimed to drastically reduce oxygen supplies (e.g., full replacement of O_2_ by N_2_). Hence our cortical slices operate at significantly higher oxygen tension levels that are unlikely to impose hypoxic conditions. (ii) The reduced ability of adult tissue to cope with lack of oxygen is usually determined by comparing it to *very* young tissue. For instance, while hippocampal slices form neonate animals exhibit significantly reduced sensitivity to anoxia, by 15do the responses are indistinguishable to those in adult slices (Cherubini et al., 1989). Similarly, *in vivo* studies have shown that the time it takes for the animals to start accumulating [K^+^]_*o*_ rapidly after nitrogen inhalation (a measure of how well they cope with reduced oxygen) is longer for animals under 12 days old, but is near adult values from 16do onwards (Hansen, 1976). At these ages most Up state parameters (occurrence, duration, Up state index) are at peak levels and begin to decline only later, in adolescence or adulthood. Therefore, there is no temporal correlation between the observed changes in Up state activity and the differential sensitivity to reduced oxygen. (iii) If the reduced activity we observe in older slices were due to metabolic constraints imposed by inadequate oxygenation, we would expect the different Up state parameters to change with a similar time course. However, as described in the Results, the different parameters exhibit distinct developmental time courses, which cannot be readily attributed to a compromised ability to cope with reduced oxygen. (iv) If the reduced activity were due to metabolic constraints, it would be expected that the Up state generating potential at those ages would have reached a plateau. However, pharmacological manipulations can significantly increase both duration and occurrence of Up states, as we recently showed after blocking endogenous nicotinic transmission (Sigalas et al., 2015). The same is true when slices are bathed in low doses of gabazine to block GABA_A_R-mediated responses, but well below the level for generating epileptiform bursts (unpublished observations). Such observations indicate that the network is capable of higher activity rates, and is unlikely to be limited by inability to cope with shortages in oxygen.

### Potential mechanisms underlying the ontogenetic changes in up state activity

The developmental progression of Up state dynamics is likely to be mechanistically complex and a full discussion of the factors that could underlie the observed changes is beyond the scope of the present study. Nevertheless, there are two observations worth making: First, the overall development of Up state activity (as reflected in both the Up state index and the % time in Up state) follows an inverted U-shape profile with a peak before adolescence and a protracted decline toward adult levels, which is highly reminiscent of the developmental profile of a number of anatomical and functional parameters in both rodents and primates, including the number of neurons and synapses, as well as brain glucose consumption (Huttenlocher, 1979, 1990; Blue and Parnavelas, 1983; Chugani et al., 1987; Micheva and Beaulieu, 1995, 1996; Huttenlocher and Dabholkar, 1997; Chugani, 1998; Bandeira et al., 2009; Petanjek et al., 2011; Bianchi et al., 2013; Herculano-Houzel et al., 2013; Ouellet and de Villers-Sidani, 2014; de Vivo et al., 2014). This is consistent with the notion that Up state activity detected in the LFP signal reflects the number of cortical neurons that enter near-synchronously the Up or the Down state, as well as the overall number and strength of the synaptic connections among them. Second, the various parameters of Up state events (e.g., size, occurrence, and spectral content) follow distinct developmental trajectories, implying that the generation and termination of spontaneous Up states are controlled by different cellular/synaptic mechanisms. This is in line with previous results (Cunningham et al., 2006; Mann et al., 2009; Fanselow and Connors, 2010; Sanchez-Vives et al., 2010) and further highlights testable predictions/hypotheses regarding the mechanisms that support these temporarily activated and self-maintained depolarized states.

In the following paragraphs we provide examples of highly suggestive correspondence between established developmental milestones and modifications of Up state parameters, as a basis for further investigating the mechanisms underlying this mode of network dynamics. For instance, the sharp decline in amplitude of the LFP signal after 10do (Figure 4B) occurs at the time the cortex undergoes a fundamental transition from a highly synchronized to a much more desynchronized state of activity (Golshani et al., 2009; Rochefort et al., 2009), as well as from a depolarizing to a hyperpolarizing action of GABA (as reviewed in Ben-Ari, 2014). Hence it is likely that a reduction in synaptic synchronization and/or excitatory drive would be reflected as a significant drop in the amplitude of the LFP signal. On the other hand, the dramatic increase and subsequent decrease in Up state occurrence during the third postnatal week and adolescence, respectively, coincides with changes in numbers of neurons and synapses during those periods (Blue and Parnavelas, 1983; Micheva and Beaulieu, 1995, 1996; De Felipe et al., 1997; Chen et al., 2014).

Moreover, the peak in the γ-power content of Up states that we observe in the 13–18do group (Figure 5I) could be associated with the maturation of fast spiking (FS) inhibitory interneurons and their connectivity (Goldberg et al., 2011; Pangratz-Fuehrer and Hestrin, 2011). γ-oscillations, a hallmark of cortical activity during sensory processing and cognition (Singer, 1993), are also present within Up states and depend on a gamma-modulated drive of FS inhibitory cells onto excitatory, regular-spiking (RS) cells (Hasenstaub et al., 2005; Morita et al., 2008; Puig et al., 2008; Fanselow and Connors, 2010). FS cells themselves exhibit intrinsic subthreshold membrane potential oscillations in the γ-frequency range (Llinás et al., 1991; Goldberg et al., 2008) and drive other cells of the neocortex in the γ-rhythm (Cardin et al., 2009). Interestingly, the intrinsic firing properties and γ-oscillations of FS cells, as well as their drive onto RS cells, increase from 10 to 18do (Goldberg et al., 2011), which could at least partially account for the significant increase in Up state γ-power that we describe during the same period and which coincides with the onset of whisking behavior (14do) (Landers and Philip Zeigler, 2006).

Finally, the decrease in Up state duration to adult levels that takes place during adolescence could be related to the maturation of somatostatin-sensitive (SOM) inhibitory neurons and/or GABA_B_ signaling. Previous studies have shown that both these factors affect the termination of Up states (Mann et al., 2009; Fanselow and Connors, 2010; Craig et al., 2013). SOM cells have been shown to mature during the third postnatal week (Kinnischtzke et al., 2012) and at the same time they become disinhibited from both synaptic and tonic inhibition (Vardya et al., 2008). Therefore, inhibition mediated by SOM cells becomes maximal at exactly the time that Up state duration begins to decrease (21do+). At the same time there is an increase in GABA_*B*_ signaling between the pre-adolescent period and adulthood (Stöhr et al., 2004). Since suppression of GABA_*B*_ receptors leads to increased Up state duration (Mann et al., 2009; Craig et al., 2013) the maturation of GABA_*B*_ signaling may well-contribute to the decrease in Up state duration that we observed during adolescence.

### Spontaneous up states as a marker of cortical regional specificity

In addition to the modulation of Up states with age, we found moderate but significant differences in spontaneously active S1BF and M1 cortical slices. This regional heterogeneity in Up state activity was anticipated on the basis of the intrinsic differences in cytoarchitecture and connectivity patterns between the two regions, such as the presence or absence of layer 4 (Donoghue and Wise, 1982), the differential interlaminar inhibitory connections (Kätzel et al., 2011), the different ratios of synaptic proteins (Pinto et al., 2013), or the flow of excitation in opposite directions (Shepherd and Svoboda, 2005; Weiler et al., 2008) reviewed in Shipp (2005) and Beul and Hilgetag (2014).

Previous studies in slices from visual cortex have indicated that Up states originate in layer 5 from where they propagate first to layer 6 and subsequently to layers 2/3 (Sanchez-Vives and McCormick, 2000). Assuming this also holds for M1 cortex, one could speculate that the lower incidence of Up states we record in the supragranular layers may reflect the absence of an intracortical ascending pathway in M1 (Weiler et al., 2008). At the same time, since the strength of local inhibition affects Up state duration (Mann et al., 2009; Sanchez-Vives et al., 2010), differences in Up state duration between S1BF and M1 cortex may result from intrinsic variations in their inhibitory networks. Notably, a recent *in vivo* study did not detect differences in either incidence or duration between Up states of M1 and S1 (although there was a trend for longer Up states in M1; (Ruiz-Mejias et al., 2011). The discrepancy between their *in vivo* and our *in vitro* results may be at least partly attributed to the propagation of Up states via long-range connections that synchronize the slow oscillation over distal parts of the cortex (Amzica and Steriade, 1995; Massimini et al., 2004), hence masking differences in local network activity.

The distinct developmental profile of Up states in M1 cortex, both in timing and intensity, was not necessarily anticipated since there is a lack of consensus in the literature regarding the comparative development of different cortical regions, with one interpretation highlighting the simultaneous maturation of distinct cortical areas (e.g., Rakic et al., 1986), and the other promoting a sequential development that proceeds in the caudal-to-rostral direction (e.g., Huttenlocher and Dabholkar, 1997). Overall, the issue seems to depend on the particular species, but also on the specific parameter that is examined. For instance, measures of synaptic density in non-human primates provide evidence of a synchronous development among cortical areas (Rakic et al., 1986; Bianchi et al., 2013), whereas estimates of dendritic morphology in the same species suggest a delayed development of the prefrontal compared to the sensorimotor cortex (Bianchi et al., 2013). On the other hand, similar work in humans points to a progression in cortical development from primary sensory to higher cortical areas (Huttenlocher, 1990; Huttenlocher and Dabholkar, 1997). The notion of sequential cortical development was recently corroborated by functional (EEG) studies that showed a caudal-to-rostral maturation of slow wave activity in humans (Kurth et al., 2010). Finally, in line with a sensory-to-motor sequence in cortical development, normal sensory experience at early developmental stages was found to be a critical requirement for normal motor output organization and performance in rats (Huntley, 1997). To our knowledge, our study is the first attempt to compare the development of intrinsic network activity in primary sensory and motor cortex in the mouse, and our results point to faster and more intense developmental changes in S1.

## Concluding remarks

In this study we propose that spontaneous Up states recorded in acute brain slices can serve as a single metric index of cortical maturation and differentiation, defining periods of intense re-organization in the underlying neuronal circuits. Although the basic network seems to be in place from the third postnatal week, the changes in Up state activity imply a maturational process in the endogenous structure, and therefore the computational capacity, of the cortical circuit up until early adulthood, implicating adolescence as a sensitive period during which cortical networks are still being stabilized. We suggest this functional index represents the maturational changes of the cortex in the framework of network dynamics, and hence provides an integrated view of cortical development across the entire life span of the mouse. We therefore propose it can be useful as a baseline against which to compare cortical dynamics in animal models of neurological and mental disorders of cortical origin. Hence this work introduces an *in vitro* model for the development and maturation of the cortical network and sets the stage for the discovery of endophenotypes for disorders that are manifested as disruptions in the excitation-inhibition balance highlighting optimal periods of therapeutic intervention.

## Author contributions

PR contributed to the conception and design of the work; the acquisition, analysis, and interpretation of data for the work; drafting the manuscript and critically revising it. DA and NL contributed to the analysis and interpretation of data, drafting the work and critically revising it. PT contributed to the analysis of data for the work. CS contributed to the acquisition and analysis of data for the work and critically revising the manuscript. IS contributed to the conception and design of the work, interpretation of data for the work, drafting the manuscript, and critically revising it.

## Funding

This work was supported by a Marie Curie Re-integration grant (INTRICA, Ref. Number 256592) to PR, and an EU FP7 RegPot grant (TRANSMED, Ref. Number 245928) to IS.

### Conflict of interest statement

The authors declare that the research was conducted in the absence of any commercial or financial relationships that could be construed as a potential conflict of interest.

## Footnotes

^1^Distance correlation is a statistical measure of dependence that generalizes the concept of linear correlation, by capturing the joint (potentially nonlinear) dependence of vectorial observations that may lie in spaces of different dimensionality. Its values range between 0 and 1, with small values denoting independence.
