# Supplementary figures and images for: Spontaneous Up states in vitro: a single-metric index of the functional maturation and regional differentiation of the cerebral cortex
Source: Front Neural Circuits. 2015 Oct 13;9:59. doi: 10.3389/fncir.2015.00059 (PMC4603250; doi:10.3389/fncir.2015.00059)

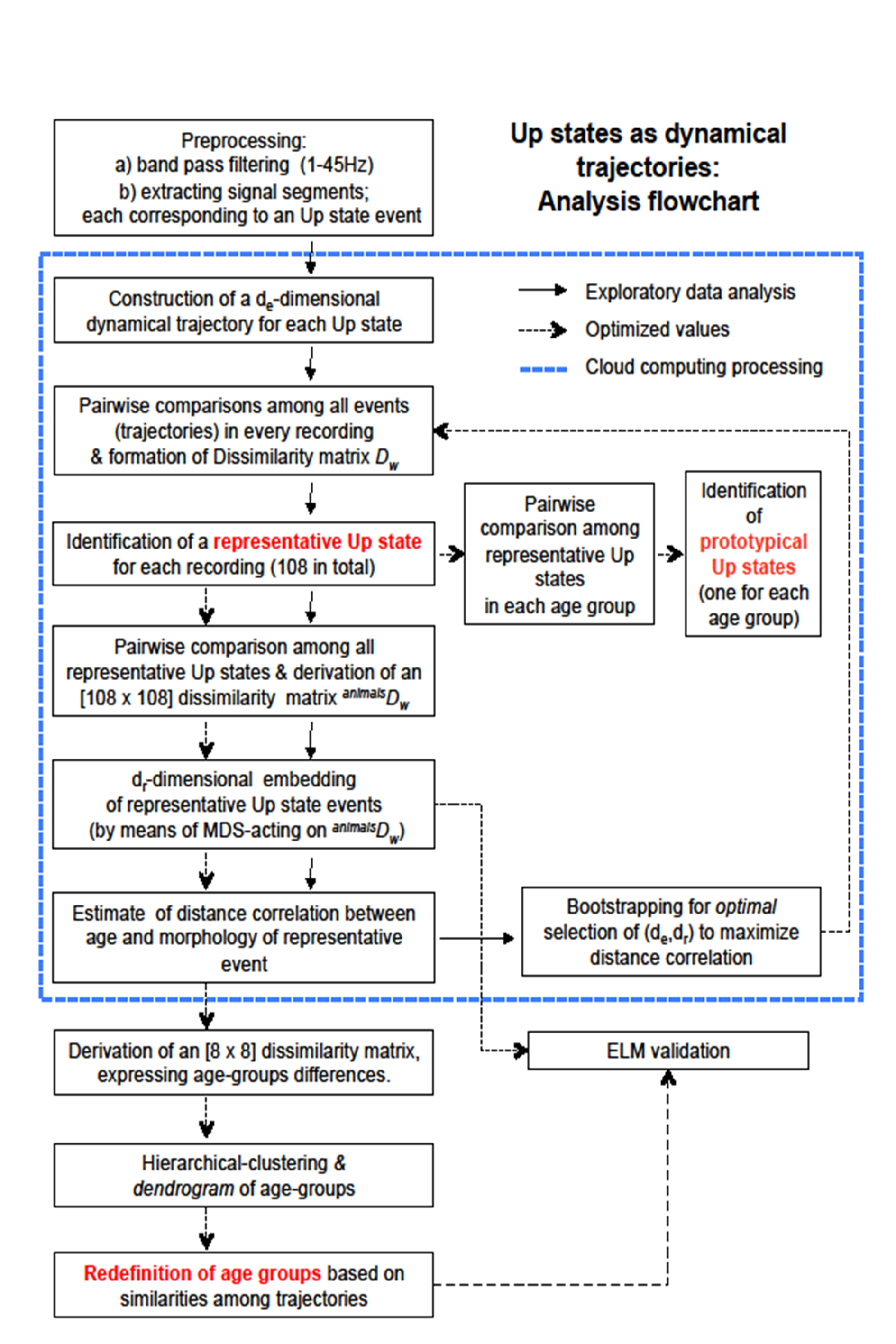

Supplement: Supplementary file 1 [file FigureS1.TIF]

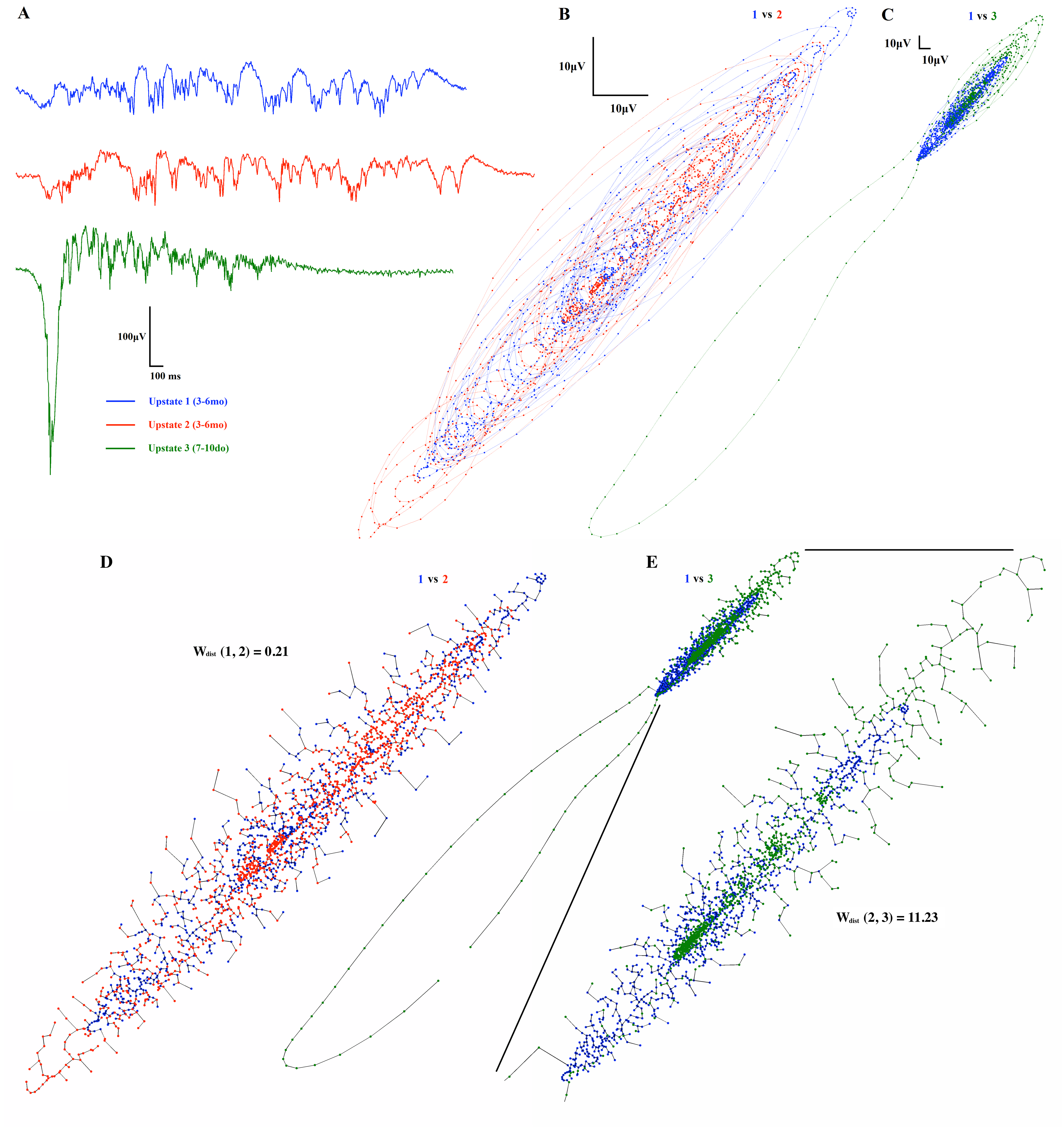

Supplement: Supplementary file 2 [file FigureS2.TIF]

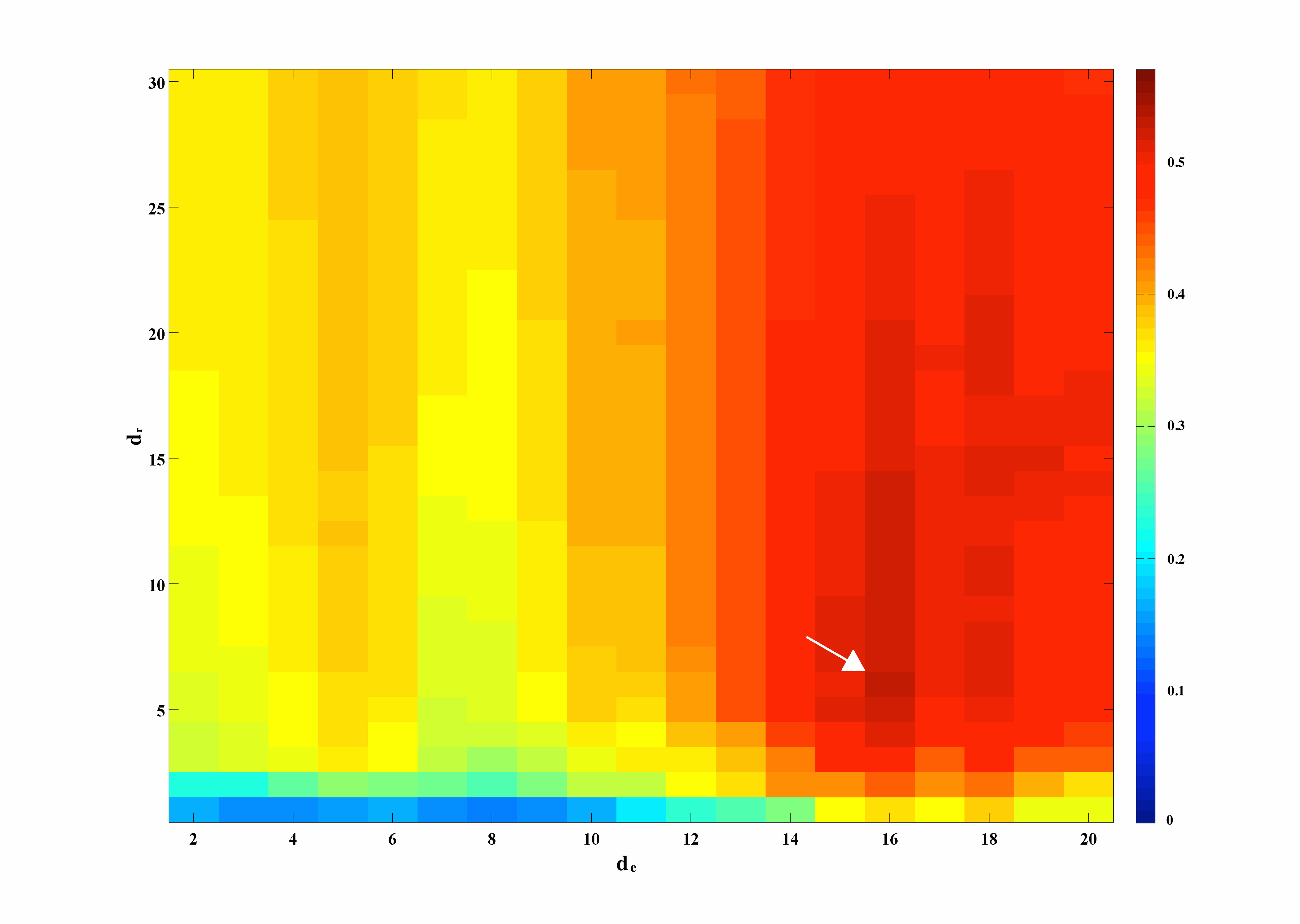

Supplement: Supplementary file 3 [file FigureS3.TIF]

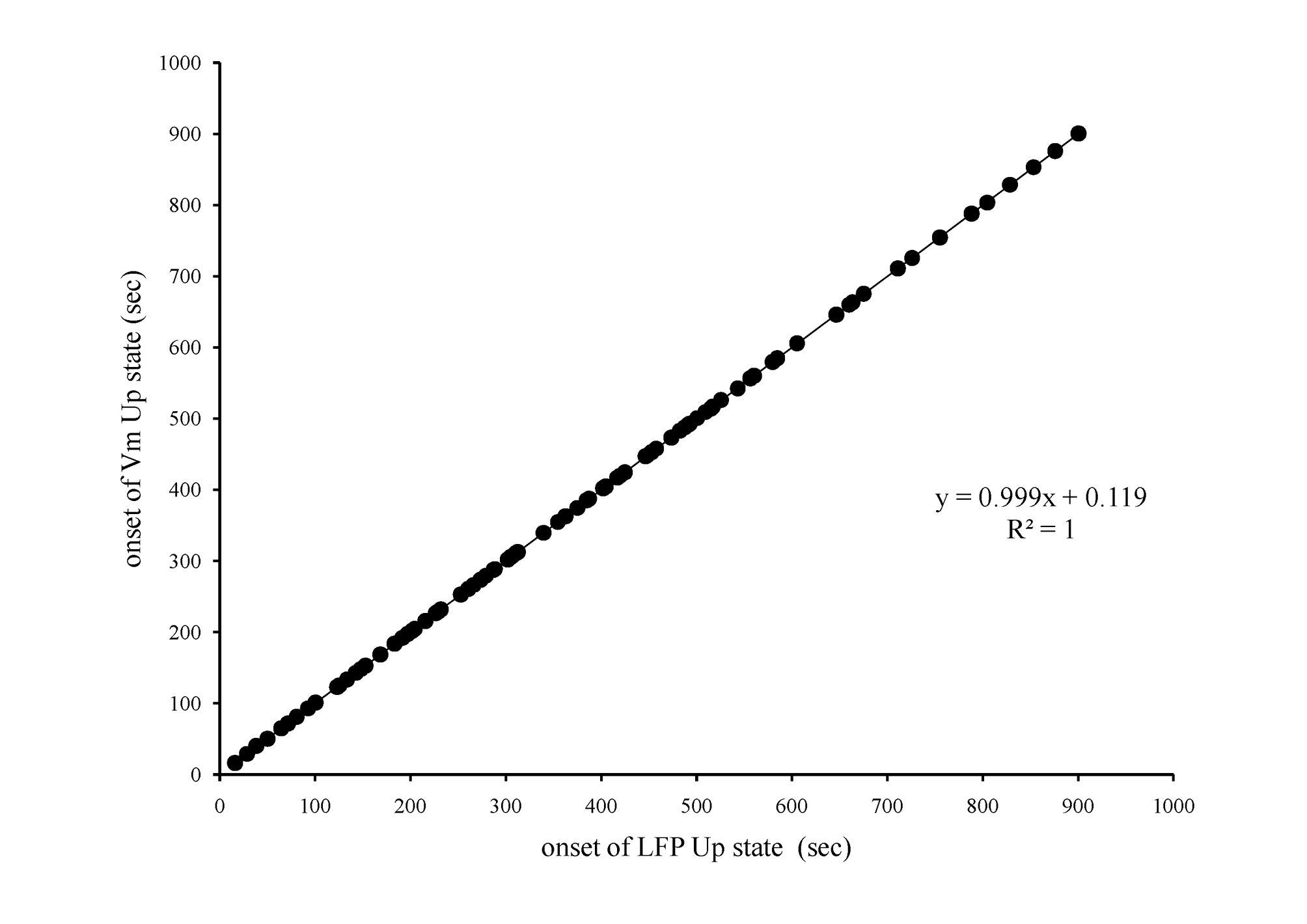

Supplement: Supplementary file 4 [file FigureS4.TIF]

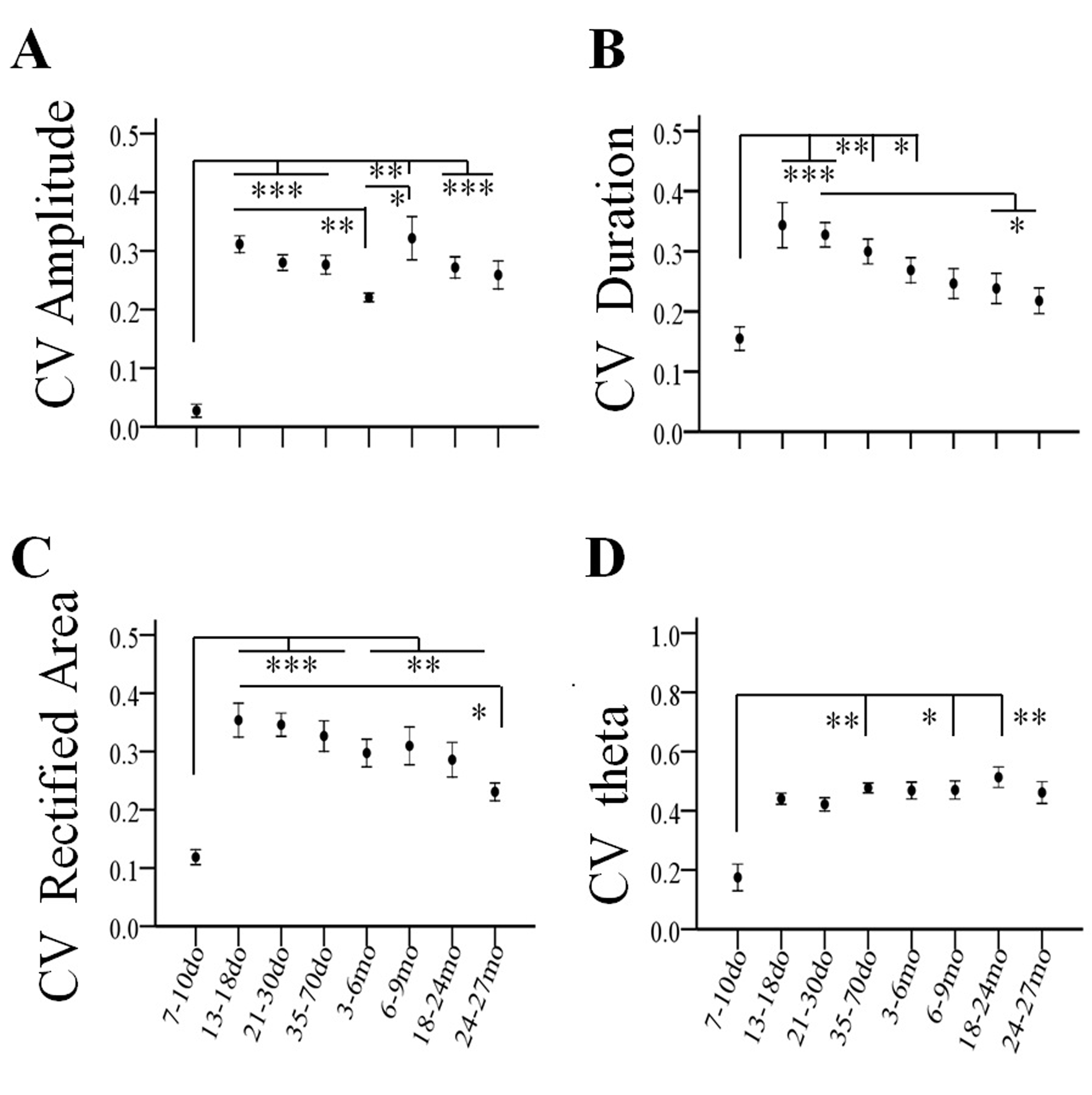

Supplement: Supplementary file 5 [file FigureS5.TIF]
