# Supplementary material for: Spontaneous Up states in vitro: a single-metric index of the functional maturation and regional differentiation of the cerebral cortex
Source: Front Neural Circuits. 2015 Oct 13;9:59. doi: 10.3389/fncir.2015.00059 (PMC4603250; doi:10.3389/fncir.2015.00059)
Supplement: Supplementary file 6 [file SupplementaryText.PDF]

SUPPLEMENTARY TEXT

SUPPLEMENTARY TEXT

I. A dissimilarity measure for dynamical trajectories based on multivariate Wald-Wolfowitz (WW) test. In this section, we first provide a short description of the WW-test (adapted from (Laskaris and Ioannides 2001)) and then demonstrate its use for expressing the dissimilarity between two given Up state waveforms.

Multivariate WW test was introduced as a non-parametric test for the *two-sample problem*; i.e. for testing if two sets of multivariate observations are coming from the same distribution (the H_o hypothesis). Given two p-dimensional point samples $\{X_i\}_{i=1:m}$ and $\{Y_i\}_{i=1:n}$ the following steps are employed: first, disregarding the sample identity of each point, the minimal spanning tree (MST) graph of the overall sample is constructed. In this graph, the points play the role of nodes and there are exactly N-1 (N=n+m) edges providing paths between every pair of nodes. Then, based on the sample identities of the points, a test statistic R is computed. R is the total number of *runs*, while a *run* is defined as a consecutive sequence of identical sample identities. Rejection of H_o occurs for small values of R. The null distribution of the test statistic is derived, based on combinatorial analysis. Based on the number of edge pairs of MST sharing a common node and the degrees of the nodes, the mean and variance of R can be calculated (see (Laskaris and Ioannides 2001)). It has been shown that the w-index,

$$w = \frac{R - E[R]}{\sqrt{Var[R]}}$$

approaches (asymptotically) the standard normal distribution. This enables the computation of the significance level (p-value) for the acceptance of the hypothesis H_o. In our case, the w-index is utilized to define a dissimilarity measure as $w_{dist} = |w|.H(-w)$, where H(x) is the Heaviside step function. The higher the value of w_{dist}, the more dissimilar the point-sets are considered to be. It should be noticed, that this measure can treat point samples of different size and has a model-free character.

To compare quantitatively two Up state events, we used their extracted signals segments *x(t)* and *y(t)* to first reconstruct the corresponding dynamics by forming the trajectories $X_t=[x(t),x(t+1),...x(t+d_e)]$ and $Y_t=[y(t),y(t+1),...y(t+d_e)]$ within a common phase-space. The two point-samples $\{X_t\}_{t=1:m}$ and $\{Y_t\}_{t=1:n}$ were then formed and the $w_{dist} = w(\{X_t\},\{Y_t\})$ was computed. Figure S2 demonstrates these steps for three Up state waveforms.

II. Optimization of *de* and *dr* parameters. We followed a bootstrapping procedure in which different settings for the d_e-d_r pair were tested, all the involved steps (from detecting representative events to analyzing them across age-groups) were implemented 100 times for each particular pair. Each time, based on random sampling with replacement, the distance-correlation between age and variations in Up state event waveforms were measured. The distribution of the obtained values was then calculated for all (d_e, d_r) pairs and used in detecting the pair associated with the systematically highest distance-correlation (Fig. S3).

III. ELM classifier for identifying the age-group associated with a given Up state waveform. ELM employs a simple learning algorithm which is based on N distinct input-output pairs (x_j, t_j) with $x_j \in R^n$ and $t_j \in R^m$ and designs a Single Hidden Layer Feedforward Network (SLFN) that reaches the smallest training error while achieving

high generalization performance. The transfer function of the network has the following compact form:

$$\sum_{i=1}^{L} \mathbf{a_i}\, g(\mathbf{w_i}.\mathbf{x_j} + b_i) = \mathbf{o_j}, \quad j = 1,\ldots,N$$

with L a user-defined number (L<<N) of hidden neurons, *g(x)* a user-defined type of activation function, $\mathbf{a_i}$ =[a_{i1} , a_{i2} ..., a_{im}] the weight vector connecting the ith hidden node with the output nodes, b_i the threshold of the corresponding hidden node and ($\boldsymbol{w_i}.\boldsymbol{x_j}$) denoting the inner-product of $\boldsymbol{w_i}$ and $\boldsymbol{x_j}$. The ELM algorithm can then be summarized within the following steps.

Step-1: Randomly assign w_i and b_i

Step-2. Calculate the hidden layer output matrix $\mathbf{H_L}$

$$\mathbf{H}_{L\,[N\times L]} = \mathbf{H}(L) = \begin{bmatrix} g(\mathbf{w}_1\mathbf{x}_1 + b_1) & \ldots & g(\mathbf{w}_1\mathbf{x}_N + b_1) \\ & \ldots & \\ g(\mathbf{w}_1\mathbf{x}_N + b_1) & \ldots & g(\mathbf{w}_L\mathbf{x}_N + b_L) \end{bmatrix}$$

Step-3. Calculate the output weight matrix

$$\mathbf{B} = \begin{bmatrix} \mathbf{a_1} \\ \vdots \\ \mathbf{a_L} \end{bmatrix} = \mathbf{H_L^*}\,\mathbf{T} = \mathbf{H_L^*} \begin{bmatrix} \mathbf{t_1} \\ \vdots \\ \mathbf{t_N} \end{bmatrix}$$

where **H*** denotes the Moore-Penrose generalized inverse of matrix **H.**

A particular implementation of ELM algorithm (Huang 2014) was employed so as to tailor the SLFN to our classification task. In particular, the output layer of the SFLN consisted of 5 'neurons' (each one was dedicated to one of the 5 age groups as detected by the dendrogram) and the activation function was of sigmoid type. The training data was in the form of pairs $\{(x_j, t_j)\}_{j=1:N}$. The input vector [$x_i \in R^{16}$] contained the image of the representative Up state waveform in the MDS-map (i.e. the corresponding coordinates), and the output vector [$t_j \in R^5$] was an ordered 5-tuple encoding the classification label (with '1' at the neuron associated with the particular age-group the animal came from, and zero elsewhere). During testing, the trained SFLN was provided with the MDS-based coordinates x_{test} (corresponding to the representative Up state waveform) as input and returned a prediction about the target vector t_{test} (i.e. the age-related label of the recording).

REFERENCES

Huang GB. 2014. MATLAB codes of ELM algorithm. http://www.ntu.edu.sg/home/egbhuang/elm_codes.html.

Laskaris NA, Ioannides AA. 2001. Exploratory data analysis of evoked response single trials based on minimal spanning tree. Clinical neurophysiology : official journal of the International Federation of Clinical Neurophysiology 112:698-712.

SUPPLEMENTARY FIGURE LEGENDS

FIGURE S1: **Up states as dynamical trajectories: analysis flow chart.** The overall methodology depends on two parameters, namely the time-delay embedding d_e and dimensionality of MDS-based embedding d_r. During this procedure, all the involved steps (left column) were implemented in an iterative fashion and optimized values for the (d_e-d_r) pair were obtained through bootstrapping (so as to maximize the distance correlation index between age and variations in Up state waveforms). Then, optimal settings were considered in order to classify (and validate through ELM) all representative Up state waveforms into *redefined* distinct age groups based on the similarities of their dynamical trajectories. Prototypical Up states for original age groups were also identified for visualization purposes only (they were not used for further analysis).

FIGURE S2. Exemplifying pairwise comparisons of Up state events based on the Wald-Wolfowitz test. (A) Individual Up states recorded from animals of different ages (3-6mo: blue and red traces; 7-10do: green trace). **(B-C)** The waveforms are represented, via time-delay embedding, as dynamical trajectories in a common phase-space (for visualization purposes only the 1st and 3rd coordinates are depicted). **(D-E)** Based on the overall MST-graph (black lines), two trajectories are considered dissimilar if their corresponding nodes are kept well separated on the graph (as in the case of trajectories 1 (blue) and 3 (green)).

FIGURE S3. Optimization of the d_e and d_r parameters based on bootstrapping. A heat map representing the mean value of distance correlation index scores for all pairs of time-delay embedding parameters ($d_e \in [1,20]$) and dimensionality values ($d_r \in [1,30]$) of MDS-based mapping. For each pair (d_e, d_r), the overall methodology (section 2.4.2) had been applied to 100 bootstrap samples (sampling with replacement) of the original data (i.e. the 108 recordings). This step included the definition of the representative waveform for each recording and the measurement of association between age and Up state trajectory, based on distance-correlation.

FIGURE S4. Correlating time of onset for LFP- and Vm-recorded spontaneous Up states. Onset times for Up states simultaneously recorded in the LFP and intracellular traces were plotted for a number of randomly selected data files (7 recordings, 87 Up states) and revealed a perfect temporal alignment between the two (linear regression with a slope of 1, R^2=1).

FIGURE S5: Age-dependent changes in the variability of Up states recorded from mouse S1BF cortex. Scatter plots illustrate average coefficient of variation (CV) values for Up state amplitude (**A**), duration (**B**), rectified area (**C**) and normalized power in the θ-band (**D**) for the eight age groups: 7-10do 13-18do, 21-30do, 35-70do, 3-6mo, 6-9mo, 18-24mo and 24-27mo. Note, the significantly lower CVs of the youngest animals, indicating that Up states in this age are highly stereotyped.
