# Supplementary material for: Spontaneous Up states in vitro: a single-metric index of the functional maturation and regional differentiation of the cerebral cortex
Source: Front Neural Circuits. 2015 Oct 13;9:59. doi: 10.3389/fncir.2015.00059 (PMC4603250; doi:10.3389/fncir.2015.00059)
Supplement: Supplementary file 7 [file Table1.PDF]

Content of Supplement Table 1

**Table 1a**	Post-hoc pairwise comparison analysis of age's significant effect on the ***occurrence*** of Up states.
**Table 1b**	Post-hoc pairwise comparison analysis of age's significant effect on the ***duration*** of Up states.
**Table 1c**	Post-hoc pairwise comparison analysis of age's significant effect on the ***amplitude*** of Up states.
**Table 1d**	Post-hoc pairwise comparison analysis of age's significant effect on the ***rectified area*** of Up states.
**Table 1e**	Post-hoc pairwise comparison analysis of age's significant effect on the ***percent (%) time*** in the Up state.
**Table 1f**	Post-hoc pairwise comparison analysis of age's significant effect on the ***Up state index***
**Table 1g**	Post-hoc pairwise comparison analysis of age's significant effect on the Up state ***normalized delta***
**Table 1h**	Post-hoc pairwise comparison analysis of age's significant effect on the Up state ***normalized theta***
**Table 1i**	Post-hoc pairwise comparison analysis of age's significant effect on the Up state ***normalized beta***
**Table 1j**	Post-hoc pairwise comparison analysis of age's significant effect on the Up state ***normalized gamma***
**Table 1k**	Post-hoc pairwise comparison analysis of age's significant effect on the Up state ***normalized low frequencies (delta+theta)***
**Table 1l**	Post-hoc pairwise comparison analysis of age's significant effect on the Up state ***normalized high frequencies (beta+gamma)***
**Table 1m**	Post-hoc pairwise comparison analysis of age's significant effect on Up state **CV interevent interval (i.e.i.)**
**Table 1n**	Post-hoc pairwise comparison analysis of age's significant effect on Up state ***CV duration***
**Table 1o**	Post-hoc pairwise comparison analysis of age's significant effect on Up state ***CV amplitude***
**Table 1p**	Post-hoc pairwise comparison analysis of age's significant effect on Up state ***CV rectified area***
**Table 1q**	Post-hoc pairwise comparison analysis of age's significant effect on Up state ***CV normalized theta power***
**Table 1r**	Post-hoc pairwise comparison analysis of age's significant effect on Up state ***CV normalized beta power***
**Table 1s**	Post-hoc pairwise comparison analysis of age's significant effect on Up state ***CV normalized gamma power***

Table 1a

Post-hoc pairwise comparison analysis of age's significant effect on the occurrence of Up states

**Age**<br>Occurrence (min^{-1})<br>(mean ±sd)	**7-10do**<br>(0.20±0.08)	**13-18do**<br>(2.18±0.67)	**21-30do**<br>(1.97±0.68)	**35-70do**<br>(1.75±1.09)	**3-6mo**<br>(0.85±0.65)	**6-9mo**<br>(0.82±0.62)	**18-24mo**<br>(0.62±0.47)	**24-27mo**<br>(0.67±0.54)
**7-10do**<br>(0.20±0.08)		***<br>q(8,100)=7.64<br>p<0.001	***<br>q(8,100)=6.57<br>p<0.001	***<br>q(8,100)=6.20<br>p<0.001	*<br>q(8,100)=3.35<br>p=0.024	n.s.<br>q(8,100)=2.82<br>p=0.101	n.s.<br>q(8,100)=2.35<br>p=0.277	n.s.<br>q(8,100)=2.36<br>p=0.271
**13-18do**<br>(2.18±0.67)			n.s.<br>q(8,100)=0.65<br>p=0.998	n.s.<br>q(8,100)=2.01<br>p=0.477	***<br>q(8,100)=5.60<br>p<0.001	***<br>q(8,100)=5.50<br>p<0.001	***<br>q(8,100)=6.59<br>p<0.001	***<br>q(8,100)=5.47<br>p<0.001
**21-30do**<br>(1.97±0.68)				n.s.<br>q(8,100)=1.15<br>p=0.943	**<br>q(8,100)=4.36<br>p=0.001	**<br>q(8,100)=4.40<br>p=0.001	***<br>q(8,100)=5.29<br>p<0.001	**<br>q(8,100)=4.47<br>p=0.001
**35-70do**<br>(1.75±1.09)					**<br>q(8,100)=3.73<br>p=0.007	**<br>q(8,100)=3.78<br>p=0.006	***<br>q(8,100)=4.81<br>p<0.001	**<br>q(8,100)=3.86<br>p=0.005
**3-6mo** (0.85±0.65)						n.s.<br>q(8,100)=0.40<br>p=1	n.s.<br>q(8,100)=1.21<br>p=0.927	n.s.<br>q(8,100)=0.75<br>p=0.995
**6-9mo** (0.82±0.62)							n.s.<br>q(8,100)=0.70<br>p=0.997	n.s.<br>q(8,100)=0.35<br>p=1
**18-24mo**<br>(0.62±0.47)								n.s.<br>q(8,100)=0.28<br>p=1

Note: All pairwise comparisons for the 8 age groups of interest (7-10do, 13-18do, 21-30do, 35-70do, 3-6mo, 6-9mo, 18-24mo and 24-27mo) were made using Tukey's honestly significant difference (HSD) test and the obtained values of the studentized range statistic (q) were compared to critical values for n=8 means and df_{error}= 100 for a significance level of 0.05. Significance is indicated as * for p<0.05, ** for p<0.01 and *** for p<0.001. Results in each cell demonstrate level of significance followed by respective q and p values.

Table 1b

Post-hoc pairwise comparison analysis of age's significant effect on the duration of Up states

Age Duration (sec) (mean ±sd)	7-10do (1.81±0.42)	13-18do (2.12±0.59)	21-30do (1.72±0.60)	35-70do (1.20±0.42)	3-6mo (1.26±0.21)	6-9mo (1.13±0.41)	18-24mo (0.93±0.19)	24-27mo (1.01±0.36)
**7-10do** (1.81±0.42)		n.s. q(8,100)=1.73 p=0.667	n.s. q(8,100)=0.45 p=1	* q(8,100)=3.49 p=0.016	* q(8,100)=3.20 p=0.037	** q(8,100)=3.73 p=0.007	*** q(8,100)=5.05 p<0.001	** q(8,100)=4.13 p=0.002
**13-18do** (2.12±0.59)			n.s. q(8,100)=2.37 p=0.268	*** q(8,100)=6.27 p<0.001	*** q(8,100)=6.01 p<0.001	*** q(8,100)=6.34 p<0.001	*** q(8,100)=8.12 p<0.001	*** q(8,100)=6.58 p<0.001
**21-30do** (1.72±0.60)				* q(8,100)=3.22 p=0.036	n.s. q(8,100)=2.91 p=0.081	* q(8,100)=3.48 p=0.017	*** q(8,100)=4.89 p<0.001	** q(8,100)=3.90 p=0.004
**35-70do** (1.20±0.42)					n.s. q(8,100)=0.44 p=1	n.s. q(8,100)=0.49 p=1	n.s. q(8,100)=1.95 p=0.523	n.s. q(8,100)=1.16 p=9.41
**3-6mo** (1.26±0.21)						n.s. q(8,100)=0.90 p=0.985	n.s. q(8,100)=2.44 p=0.236	n.s. q(8,100)=1.56 p=0.774
**6-9mo** (1.13±0.41)							n.s. q(8,100)=1.32 p=0.887	n.s q(8,100)=0.67 p=1
**18-24mo** (0.93±0.19)								n.s. q(8,100)=0.51 p=1

Note: All pairwise comparisons for the 8 age groups of interest (7-10do, 13-18do, 21-30do, 35-70do, 3-6mo, 6-9mo, 18-24mo and 24-27mo) were made using Tukey's honestly significant difference (HSD) test and the obtained values of the studentized range statistic (q) were compared to critical values for n=8 means and df_{error}= 100 for a significance level of 0.05. Significance is indicated as * for p<0.05, ** for p<0.01 and *** for p<0.001. Results in each cell demonstrate level of significance followed by respective q and p values.

Table 1c

Post-hoc pairwise comparison analysis of age's significant effect on the amplitude of Up states

Age<br>Amplitude (µV)<br>(mean ±sd)	**7-10do**<br>(-184±39)	**13-18do**<br>(-86±26)	**21-30do**<br>(-74±37)	**35-70do**<br>(-57±19)	**3-6mo**<br>(-55±25)	**6-9mo**<br>(-68±30)	**18-24mo**<br>(-61±27)	**24-27mo**<br>(-71±39)
**7-10do**<br>(-184±39)		n.s.<br>q(8,100)=2.40<br>p=0.253	*<br>q(8,100)=3.39<br>p=0.021	***<br>q(8,100)=4.96<br>p<0.001	***<br>q(8,100)=5.49<br>p<0.001	**<br>q(8,100)=4.00<br>p=0.003	***<br>q(8,100)=4.80<br>p<0.001	**<br>q(8,100)=3.71<br>p=0.008
**13-18do**<br>(-86±26)			n.s.<br>q(8,100)=1.32<br>p=0.890	n.s.<br>q(8,100)=2.95<br>p=0.073	*<br>q(8,100)=3.55<br>p=0.013	n.s.<br>q(8,100)=1.90<br>p=0.554	n.s.<br>q(8,100)=2.76<br>p=0.118	n.s.<br>q(8,100)=1.69<br>p=0.696
**21-30do**<br>(-74±37)				n.s.<br>q(8,100)=1.30<br>p=0.896	n.s.<br>q(8,100)=1.80<br>p=0.619	n.s.<br>q(8,100)=0.44<br>p=1	n.s.<br>q(8,100)=1.13<br>p=0.949	n.s.<br>q(8,100)=0.34<br>p=1
**35-70do**<br>(-57±19)					n.s.<br>q(8,100)=0.56<br>p=0.999	n.s.<br>q(8,100)=0.90<br>p=0.985	n.s.<br>q(8,100)=0.20<br>p=1	n.s.<br>q(8,100)=0.92<br>p=0.983
**3-6mo**<br>(-55±25)						n.s.<br>q(8,100)=1.44<br>p=0.837	n.s.<br>q(8,100)=0.77<br>p=0.994	n.s.<br>q(8,100)=1.41<br>p=0.849
**6-9mo**<br>(-68±30)							n.s.<br>q(8,100)=0.71<br>p=0.996	n.s.<br>q(8,100)=0.08<br>p=1
**18-24mo**<br>(-61±27)								n.s.<br>q(8,100)=0.74<br>p=0.995

Note: All pairwise comparisons for the 8 age groups of interest (7-10do, 13-18do, 21-30do, 35-70do, 3-6mo, 6-9mo, 18-24mo and 24-27mo) were made using Tukey's honestly significant difference (HSD) test and the obtained values of the studentized range statistic (q) were compared to critical values for n=8 means and df_{error}= 100 for a significance level of 0.05. Significance is indicated as * for p<0.05, ** for p<0.01 and *** for p<0.001. Results in each cell demonstrate level of significance followed by respective q and p values.

Table 1d

Post-hoc pairwise comparison analysis of age's significant effect on the rectified area of Up states

Age<br>Rectified Area (μV^2)<br>(mean ±sd)	**7-10do**<br>(0.34±0.14)	**13-18do**<br>(0.21±0.09)	**21-30do**<br>(0.14±0.10)	**35-70do**<br>(0.11±0.08)	**3-6mo**<br>(0.13±0.06)	**6-9mo**<br>(0.11±0.05)	**18-24mo**<br>(0.10±0.04)	**24-27mo**<br>(0.08±0.03)
**7-10do**<br>(0.34±0.14)		**<br>q(8,100)=3.90<br>p=0.004	***<br>q(8,100)=5.74<br>p<0.001	***<br>q(8,100)=7.41<br>p<0.001	***<br>q(8,100)=6.93<br>p<0.001	***<br>q(8,100)=7.06<br>p<0.001	***<br>q(8,100)=7.58<br>p<0.001	***<br>q(8,100)=7.65<br>p<0.001
**13-18do**<br>(0.21±0.09)			n.s.<br>q(8,100)=2.41<br>p=0.250	**<br>q(8,100)=4.01<br>p=0.003	*<br>q(8,100)=3.39<br>p=0.022	**<br>q(8,100)=3.75<br>p=0.007	**<br>q(8,100)=4.21<br>p=0.001	***<br>q(8,100)=4.59<br>p<0.001
**21-30do**<br>(0.14±0.10)				n.s.<br>q(8,100)=1.13<br>p=0.948	n.s.<br>q(8,100)=0.50<br>p=1	n.s.<br>q(8,100)=1.06<br>p=0.963	n.s.<br>q(8,100)=1.32<br>p=0.891	n.s.<br>q(8,100)=2.02<br>p=0.475
**35-70do**<br>(0.11±0.08)					n.s.<br>q(8,100)=0.76<br>p=0.995	n.s.<br>q(8,100)=0.01<br>p=1	n.s.<br>q(8,100)=0.21<br>p=1	n.s.<br>q(8,100)=1.13<br>p=0.948
**3-6mo**<br>(0.13±0.06)						n.s.<br>q(8,100)=0.69<br>p=0.997	n.s.<br>q(8,100)=0.98<br>p=0.976	n.s.<br>q(8,100)=1.81<br>p=0.617
**6-9mo**<br>(0.11±0.05)							n.s.<br>q(8,100)=0.21<br>p=1	n.s.<br>q(8,100)=1.08<br>p=0.959
**18-24mo**<br>(0.10±0.04)								n.s.<br>q(8,100)=0.95<br>p=0.980

Note: All pairwise comparisons for the 8 age groups of interest (7-10do, 13-18do, 21-30do, 35-70do, 3-6mo, 6-9mo, 18-24mo and 24-27mo) were made using Tukey's honestly significant difference (HSD) test and the obtained values of the studentized range statistic (q) were compared to critical values for n=8 means and df_{error}= 100 for a significance level of 0.05. Significance is indicated as * for p<0.05, ** for p<0.01 and *** for p<0.001. Results in each cell demonstrate level of significance followed by respective q and p values.

Table 1e

Post-hoc pairwise comparison analysis of age's significant effect on the percent (%) time in the Up state

Age<br>% time Up state<br>(mean ±sd)	**7-10do**<br>(0.60±0.25)	**13-18do**<br>(7.41±2.56)	**21-30do**<br>(5.41±3.01)	**35-70do**<br>(3.49±2.73)	**3-6mo**<br>(1.77±1.30)	**6-9mo**<br>(1.75±1.50)	**18-24mo**<br>(0.99±0.80)	**24-27mo**<br>(1.23±1.09)
**7-10do**<br>(0.60±0.25)		***<br>$\chi^2(1)$=88.93<br>p< 10^{-5}	***<br>$\chi^2(1)$=40.69<br>p< 10^{-5}	***<br>$\chi^2(1)$=45.56<br>p< 10^{-5}	***<br>$\chi^2(1)$=20.63<br>p< 10^{-5}	*<br>$\chi^2(1)$=11.56<br>p=0.0007	n.s.<br>$\chi^2(1)$=5<br>p=0.025	n.s.<br>$\chi^2(1)$=2.88<br>p=0.09
**13-18do**<br>(7.41±2.56)			**<br>$\chi^2(1)$=15.29<br>p=0.00009	***<br>$\chi^2(1)$=103<br>p< 10^{-5}	***<br>$\chi^2(1)$=36.03<br>p< 10^{-5}	***<br>$\chi^2(1)$=54.23<br>p< 10^{-5}	***<br>$\chi^2(1)$=65.63<br>p< 10^{-5}	***<br>$\chi^2(1)$=72.01<br>p< 10^{-5}
**21-30do**<br>(5.41±3.01)				n.s.<br>$\chi^2(1)$=0.221<br>p=0.638	n.s.<br>$\chi^2(1)$=21.30<br>p=0.024	**<br>$\chi^2(1)$=14.52<br>p=0.00014	***<br>$\chi^2(1)$=22.04<br>p< 10^{-5}	***<br>$\chi^2(1)$=26.73<br>p< 10^{-5}
**35-70do**<br>(3.49±2.73)					n.s.<br>$\chi^2(1)$=7.348<br>p=0.007	***<br>$\chi^2(1)$=18.05<br>p=0.00002	***<br>$\chi^2(1)$=26.16<br>p< 10^{-5}	***<br>$\chi^2(1)$=31.11<br>p< 10^{-5}
**3-6mo**<br>(1.77±1.30)						n.s.<br>$\chi^2(1)$=2.667<br>p=0.102	n.s.<br>$\chi^2(1)$=6.750<br>p=0.009	*<br>$\chi^2(1)$=9.80<br>p=0.0017
**6-9mo**<br>(1.75±1.50)							n.s.<br>$\chi^2(1)$=1<br>p=0.317	n.s.<br>$\chi^2(1)$=2.46<br>p=0.117
**18-24mo**<br>(0.99±0.80)								n.s.<br>$\chi^2(1)$=0.333<br>p=0.564

Note: All pairwise comparisons for the 8 age groups of interest (7-10do, 13-18do, 21-30do, 35-70do, 3-6mo, 6-9mo, 18-24mo and 24-27mo) were made using chi-square (χ^2) test for independence. In order to control for multiple comparisons levels of significance (α) of 0.05, 0.01 and 0.001 were adjusted to 0.002, 0.0004 and0.00004 , respectively (i.e. α/ number of comparison (28)). Therefore significance is indicated as * for p<0.002, ** for p<0.0004 and *** for p<0.00004. Results in each cell demonstrate level of significance followed by respective $\chi 2$ values (for df=1) and p values.

Table 1f

Post-hoc pairwise comparison analysis of age's significant effect on the Up state index

**Age**<br>Up state index<br>(mean ±sd)	**7-10do**<br>(0.07±0.04)	**13-18do**<br>(0.47±0.23)	**21-30do**<br>(0.27±0.18)	**35-70do**<br>(0.17±0.15)	**3-6mo**<br>(0.09±0.05)	**6-9mo**<br>(0.10±0.09)	**18-24mo**<br>(0.07±0.06)	**24-27mo**<br>(0.04±0.03)
**7-10do** (0.07±0.04)		***<br>q(8,100)=5.14<br>p<0.001	*<br>q(8,100)=3.15<br>p=0.043	n.s.<br>q(8,100)=2.10<br>p=0.421	n.s.<br>q(8,100)=0.59<br>p=0.999	n.s.<br>q(8,100)=0.21<br>p=1	n.s.<br>q(8,100)=0.65<br>p=0.998	n.s.<br>q(8,100)=1.47<br>p=0.819
**13-18do**<br>(0.47±0.23)			n.s.<br>q(8,100)=1.90<br>p=0.557	*<br>q(8,100)=3.82<br>p=0.005	***<br>q(8,100)=5.76<br>p<0.001	***<br>q(8,100)=5.68<br>p<0.001	***<br>q(8,100)=7.09<br>p<0.001	***<br>q(8,100)=7.20<br>p<0.001
**21-30do**<br>(0.27±0.18)				n.s.<br>q(8,100)=1.49<br>p=0.810	*<br>q(8,100)=3.19<br>p=0.039	*<br>q(8,100)=3.33<br>p=0.026	**<br>q(8,100)=4.45<br>p=0.001	***<br>q(8,100)=4.91<br>p<0.001
**35-70do**<br>(0.17±0.15)					n.s.<br>q(8,100)=1.95<br>p=0.521	n.s.<br>q(8,100)=2.19<br>p=0.366	*<br>q(8,100)=3.44<br>p=0.0.18	**<br>q(8,100)=4.02<br>p=0.003
**3-6mo** (0.09±0.05)						n.s.<br>q(8,100)=0.43<br>p=1	n.s.<br>q(8,100)=1.59<br>p=0.757	n.s.<br>q(8,100)=2.43<br>p=0.238
**6-9mo** (0.10±0.09)							n.s.<br>q(8,100)=1.01<br>p=0.971	n.s.<br>q(8,100)=1.89<br>p=0.564
**18-24mo**<br>(0.07±0.06)								n.s.<br>q(8,100)=1.05<br>p=0.965

Note: All pairwise comparisons for the 8 age groups of interest (7-10do, 13-18do, 21-30do, 35-70do, 3-6mo, 6-9mo, 18-24mo and 24-27mo) were made using Tukey's honestly significant difference (HSD) test and the obtained values of the studentized range statistic (q) were compared to critical values for n=8 means and df_{error}= 100 for a significance level of 0.05. Significance is indicated as * for p<0.05, ** for p<0.01 and *** for p<0.001. Results in each cell demonstrate level of significance followed by respective q and p values.

Table 1g

Post-hoc pairwise comparison analysis of age's significant effect on the Up state normalized delta

Age<br>Normalized delta<br>(mean ±sd)	**7-10do**<br>(0.48±0.22)	**13-18do**<br>(0.42±0.09)	**21-30do**<br>(0.45±0.10)	**35-70do**<br>(0.49±0.11)	**3-6mo**<br>(0.56±0.13)	**6-9mo**<br>(0.51±0.12)	**18-24mo**<br>(0.56±0.13)	**24-27mo**<br>(0.61±0.16)
**7-10do**<br>(0.48±0.22)		n.s.<br>q(8,100)=1.03<br>p=0.969	n.s.<br>q(8,100)=0.53<br>p=0.999	n.s.<br>q(8,100)=0.09<br>p=1	n.s.<br>q(8,100)=1.30<br>p=0.897	n.s.<br>q(8,100)=0.43<br>p=1	n.s.<br>q(8,100)=1.38<br>p=0.866	n.s.<br>q(8,100)=2.05<br>p=0.456
**13-18do**<br>(0.42±0.09)			n.s.<br>q(8,100)=0.49<br>p=1	n.s.<br>q(8,100)=1.36<br>p=0.871	n.s.<br>q(8,100)=2.85<br>p=0.095	n.s.<br>q(8,100)=1.68<br>p=0.699	n.s.<br>q(8,100)=2.89<br>p=0.085	*<br>q(8,100)=3.44<br>p=0.018
**21-30do**<br>(0.45±0.10)				n.s.<br>q(8,100)=0.72<br>p=0.996	n.s.<br>q(8,100)=2.04<br>p=0.459	n.s.<br>q(8,100)=1.05<br>p=0.965	n.s.<br>q(8,100)=2.11<br>p=0.416	n.s.<br>q(8,100)=2.73<br>p=0.125
**35-70do**<br>(0.49±0.11)					n.s.<br>q(8,100)=1.53<br>p=0.790	n.s.<br>q(8,100)=0.42<br>p=1	n.s.<br>q(8,100)=1.61<br>p=0.743	n.s.<br>q(8,100)=2.34<br>p=0.281
**3-6mo** (0.56±0.13)						n.s.<br>q(8,100)=0.99<br>p=0.975	n.s.<br>q(8,100)=0.13<br>p=1	n.s.<br>q(8,100)=1.08<br>p=0.959
**6-9mo** (0.51±0.12)							n.s.<br>q(8,100)=1.08<br>p=0.960	n.s.<br>q(8,100)=1.85<br>p=0.587
**18-24mo**<br>(0.56±0.13)								n.s.<br>q(8,100)=0.96<br>p=0.979

Note: All pairwise comparisons for the 8 age groups of interest (7-10do, 13-18do, 21-30do, 35-70do, 3-6mo, 6-9mo, 18-24mo and 24-27mo) were made using Tukey's honestly significant difference (HSD) test and the obtained values of the studentized range statistic (q) were compared to critical values for n=8 means and df_{error}= 100 for a significance level of 0.05. Significance is indicated as * for p<0.05, ** for p<0.01 and *** for p<0.001. Results in each cell demonstrate level of significance followed by respective q and p values.

Table 1h

Post-hoc pairwise comparison analysis of age's significant effect on the Up state normalized theta

Age<br>Normalized theta<br>(mean ±sd)	7-10do<br>(0.28±0.10)	13-18do<br>(0.17±0.03)	21-30do<br>(0.20±0.04)	35-70do<br>(0.20±0.04)	3-6mo<br>(0.17±0.04)	6-9mo<br>(0.20±0.04)	18-24mo<br>(0.17±0.04)	24-27mo<br>(0.15±0.05)
**7-10do**<br>(0.28±0.10)		*<br>q(8,100)=3.36<br>p=0.023	n.s.<br>q(8,100)=1.87<br>p=0.572	n.s.<br>q(8,100)=1.60<br>p=0.751	*<br>q(8,100)=3.47<br>p=0.017	n.s.<br>q(8,100)=1.62<br>p=0.736	*<br>q(8,100)=3.44<br>p=0.018	**<br>q(8,100)=3.69<br>p=0.008
**13-18do**<br>(0.17±0.03)			n.s.<br>q(8,100)=1.45<br>p=0.829	n.s.<br>q(8,100)=2.23<br>p=0.341	n.s.<br>q(8,100)=0.08<br>p=1	n.s.<br>q(8,100)=1.98<br>p=0.502	n.s.<br>q(8,100)=0.04<br>p=1	n.s.<br>q(8,100)=0.63<br>p=0.998
**21-30do**<br>(0.20±0.04)				n.s.<br>q(8,100)=0.51<br>p=1	n.s.<br>q(8,100)=1.47<br>p=0.821	n.s.<br>q(8,100)=0.38<br>p=01	n.s.<br>q(8,100)=1.48<br>p=0.818	n.s.<br>q(8,100)=1.93<br>p=0.533
**35-70do**<br>(0.20±0.04)					n.s.<br>q(8,100)=2.33<br>p=0.286	n.s.<br>q(8,100)=0.12<br>p=1	n.s.<br>q(8,100)=2.31<br>p=0.299	n.s.<br>q(8,100)=2.68<br>p=0.141
**3-6mo**<br>(0.17±0.04)						n.s.<br>q(8,100)=2.04<br>p=0.460	n.s.<br>q(8,100)=0.04<br>p=1	n.s.<br>q(8,100)=0.74<br>p=0.996
**6-9mo**<br>(0.20±0.04)							n.s.<br>q(8,100)=2.03<br>p=0.467	n.s.<br>q(8,100)=2.43<br>p=0.237
**18-24mo**<br>(0.17±0.04)								n.s.<br>q(8,100)=0.69<br>p=0.997

Note: All pairwise comparisons for the 8 age groups of interest (7-10do, 13-18do, 21-30do, 35-70do, 3-6mo, 6-9mo, 18-24mo and 24-27mo) were made using Tukey's honestly significant difference (HSD) test and the obtained values of the studentized range statistic (q) were compared to critical values for n=8 means and df_{error}= 100 for a significance level of 0.05. Significance is indicated as * for p<0.05, ** for p<0.01 and *** for p<0.001. Results in each cell demonstrate level of significance followed by respective q and p values.

Table 1i

Post-hoc pairwise comparison analysis of age's significant effect on the Up state normalized beta

Age<br>Normalized beta<br>(mean ±sd)	**7-10do**<br>(0.09±0.08)	**13-18do**<br>(0.17±0.05)	**21-30do**<br>(0.16±0.06)	**35-70do**<br>(0.14±0.06)	**3-6mo**<br>(0.11±0.04)	**6-9mo**<br>(0.11±0.04)	**18-24mo**<br>(0.11±0.05)	**24-27mo**<br>(0.09±0.05)
**7-10do**<br>(0.09±0.08)		*<br>q(8,100)=3.21<br>p=0.036	n.s.<br>q(8,100)=2.75<br>p=0.120	n.s.<br>q(8,100)=1.91<br>p=0.550	n.s.<br>q(8,100)=0.67<br>p=0.998	n.s.<br>q(8,100)=0.85<br>p=0.990	n.s.<br>q(8,100)=0.99<br>p=0.976	n.s.<br>q(8,100)=0.09<br>p=1
**13-18do**<br>(0.17±0.05)			n.s.<br>q(8,100)=0.28<br>p=1	n.s.<br>q(8,100)=1.68<br>p=0.701	*<br>q(8,100)=3.24<br>p=0.033	n.s.<br>q(8,100)=2.71<br>p=0.133	n.s.<br>q(8,100)=2.77<br>p=0.114	*<br>q(8,100)=3.54<br>p=0.014
**21-30do**<br>(0.16±0.06)				n.s.<br>q(8,100)=1.22<br>p=0.923	n.s.<br>q(8,100)=2.62<br>p=0.161	n.s.<br>q(8,100)=2.20<br>p=0.362	n.s.<br>q(8,100)=2.22<br>p=0.352	n.s.<br>q(8,100)=3.01<br>p=0.063
**35-70do**<br>(0.14±0.06)					n.s.<br>q(8,100)=1.61<br>p=0.746	n.s.<br>q(8,100)=1.18<br>p=0.934	n.s.<br>q(8,100)=1.15<br>p=0.943	n.s.<br>q(8,100)=2.15<br>p=0.388
**3-6mo**<br>(0.11±0.04)						n.s.<br>q(8,100)=0.28<br>p=1	n.s.<br>q(8,100)=0.42<br>p=1	n.s.<br>q(8,100)=0.83<br>p=0.991
**6-9mo**<br>(0.11±0.04)							n.s.<br>q(8,100)=0.11<br>p=1	n.s.<br>q(8,100)=1.00<br>p=0.973
**18-24mo**<br>(0.11±0.05)								n.s.<br>q(8,100)=1.16<br>p=0.940

Note: All pairwise comparisons for the 8 age groups of interest (7-10do, 13-18do, 21-30do, 35-70do, 3-6mo, 6-9mo, 18-24mo and 24-27mo) were made using Tukey's honestly significant difference (HSD) test and the obtained values of the studentized range statistic (q) were compared to critical values for n=8 means and df_{error}= 100 for a significance level of 0.05. Significance is indicated as * for p<0.05, ** for p<0.01 and *** for p<0.001. Results in each cell demonstrate level of significance followed by respective q and p values.

Table 1j

Post-hoc pairwise comparison analysis of age's significant effect on the Up state normalized gamma

**Age**<br>Normalized gamma<br>(mean ±sd)	**7-10do**<br>(0.03±0.02)	**13-18do**<br>(0.16±0.06)	**21-30do**<br>(0.11±0.06)	**35-70do**<br>(0.09±0.06)	**3-6mo**<br>(0.09±0.07)	**6-9mo**<br>(0.10±0.05)	**18-24mo**<br>(0.09±0.07)	**24-27mo**<br>(0.09±0.08)
**7-10do** (0.03±0.02)		***<br>q(8,100)=5.37<br>p<0.001	*<br>q(8,100)=3.35<br>p=0.024	*<br>q(8,100)=3.14<br>p=0.044	n.s.<br>q(8,100)=2.92<br>p=0.08	*<br>q(8,100)=3.56<br>p=0.013	n.s.<br>q(8,100)=2.79<br>p=0.110	n.s.<br>q(8,100)=2.21<br>p=0.354
**13-18do** (0.16±0.06)			n.s.<br>q(8,100)=1.91<br>p=0.546	n.s.<br>q(8,100)=2.86<br>p=0.091	*<br>q(8,100)=3.27<br>p=0.031	n.s.<br>q(8,100)=2.04<br>p=0.463	*<br>q(8,100)=3.29<br>p=0.029	*<br>q(8,100)=3.22<br>p=0.035
**21-30do** (0.11±0.06)				n.s.<br>q(8,100)= 0.61<br>p=0.999	n.s.<br>q(8,100)= 0.92<br>p=1	n.s.<br>q(8,100)= 0.02<br>p=0.997	n.s.<br>q(8,100)=0.99<br>p=1	n.s.<br>q(8,100)=1.21<br>p=0.995
**35-70do** (0.09±0.06)					n.s.<br>q(8,100)=0.35<br>p=1	n.s.<br>q(8,100)=0.68<br>p=0.970	n.s.<br>q(8,100)=0.45<br>p=1	n.s.<br>q(8,100)=0.75<br>p=0.995
**3-6mo** (0.09±0.07)						n.s.<br>q(8,100)=1.02<br>p=0.970	n.s.<br>q(8,100)=0.11<br>p=1	n.s.<br>q(8,100)=0.46<br>p=1
**6-9mo** (0.10±0.05)							n.s.<br>q(8,100)=1.09<br>p=0.957	n.s.<br>q(8,100)=1.30<br>p=0.895
**18-24mo** (0.09±0.07)								n.s.<br>q(8,100)=0.37<br>p=1

Note: All pairwise comparisons for the 8 age groups of interest (7-10do, 13-18do, 21-30do, 35-70do, 3-6mo, 6-9mo, 18-24mo and 24-27mo) were made using Tukey's honestly significant difference (HSD) test and the obtained values of the studentized range statistic (q) were compared to critical values for n=8 means and df_{error}= 100 for a significance level of 0.05. Significance is indicated as * for p<0.05, ** for p<0.01 and *** for p<0.001. Results in each cell demonstrate level of significance followed by respective q and p values.

Table 1k

Post-hoc pairwise comparison analysis of age's significant effect on the Up state normalized low frequencies (delta+theta)

Age<br>Normalized lower frequencies<br>(mean ±sd)	**7-10do**<br>(0.80±0.20)	**13-18do**<br>(0.60±0.10)	**21-30do**<br>(0.60±0.10)	**35-70do**<br>(0.70±0.10)	**3-6mo**<br>(0.70±0.10)	**6-9mo**<br>(0.70±0.10)	**18-24mo**<br>(0.70±0.10)	**24-27mo**<br>(0.80±0.10)
**7-10do**<br>(0.80±0.20)		*<br>q(8,100)=3.27<br>p=0.031	n.s.<br>q(8,100)=2.02<br>p=0.476	n.s.<br>q(8,100)=1.43<br>p=0.840	n.s.<br>q(8,100)=0.72<br>p=0.996	n.s.<br>q(8,100)=1.07<br>p=0.961	n.s.<br>q(8,100)=0.71<br>p=0.996	n.s.<br>q(8,100)=0.04<br>p=1
**13-18do**<br>(0.60±0.10)			n.s.<br>q(8,100)=1.19<br>p=0.934	n.s.<br>q(8,100)=2.31<br>p=0.298	*<br>q(8,100)=3.25<br>p=0.033	n.s.<br>q(8,100)=2.51<br>p=0.202	*<br>q(8,100)=3.17<br>p=0.041	*<br>q(8,100)=3.55<br>p=0.013
**21-30do**<br>(0.60±0.10)				n.s.<br>q(8,100)=0.86<br>p=0.989	n.s.<br>q(8,100)=1.67<br>p=0.707	n.s.<br>q(8,100)=1.13<br>p=0.948	n.s.<br>q(8,100)=1.64<br>p=0.728	n.s.<br>q(8,100)=2.19<br>p=0.369
**35-70do**<br>(0.70±0.10)					n.s.<br>q(8,100)=0.93<br>p=0.983	n.s.<br>q(8,100)=0.36<br>p=1	n.s.<br>q(8,100)=0.90<br>p=0.985	n.s.<br>q(8,100)=1.59<br>p=0.752
**3-6mo**<br>(0.70±0.10)						n.s.<br>q(8,100)=0.49<br>p=1	n.s.<br>q(8,100)=0.00<br>p=1	n.s.<br>q(8,100)=0.83<br>p=0.991
**6-9mo**<br>(0.70±0.10)							n.s.<br>q(8,100)=0.48<br>p=1	n.s.<br>q(8,100)=1.19<br>p=0.932
**18-24mo**<br>(0.70±0.10)								n.s.<br>q(8,100)=0.82<br>p=0.992

Note: All pairwise comparisons for the 8 age groups of interest (7-10do, 13-18do, 21-30do, 35-70do, 3-6mo, 6-9mo, 18-24mo and 24-27mo) were made using Tukey's honestly significant difference (HSD) test and the obtained values of the studentized range statistic (q) were compared to critical values for n=8 means and df_{error}= 100 for a significance level of 0.05. Significance is indicated as * for p<0.05, ** for p<0.01 and *** for p<0.001. Results in each cell demonstrate level of significance followed by respective q and p values.

Table 1l

Post-hoc pairwise comparison analysis of age's significant effect on the Up state normalized high frequencies (beta+gamma)

**Age**<br>Normalized high frequencies<br>(mean ±sd (a.u.))	**7-10do**<br>(0.13±0.09)	**13-18do**<br>(0.33±0.11)	**21-30do**<br>(0.27±0.12)	**35-70do**<br>(0.23±0.11)	**3-6mo**<br>(0.20±0.10)	**6-9mo**<br>(0.21±0.09)	**18-24mo**<br>(0.21±0.11)	**24-27mo**<br>(0.18±0.12)
**7-10do**<br>(0.13±0.09)		**<br>q(8,100)=4.31<br>p=0.001	n.s.<br>q(8,100)=2.97<br>p=0.071	*<br>q(8,100)=2.42<br>p=0.243	n.s.<br>q(8,100)=1.81<br>p=0.617	n.s.<br>q(8,100)=2.10<br>p=0.421	n.s.<br>q(8,100)=1.84<br>p=0.592	n.s.<br>q(8,100)=0.92<br>p=0.983
**13-18do**<br>(0.33±0.11)			n.s.<br>q(8,100)=1.22<br>p=0.924	n.s.<br>q(8,100)=2.42<br>p=0.243	*<br>q(8,100)=3.27<br>p=0.031	n.s.<br>q(8,100)=2.51<br>p=0.203	*<br>q(8,100)=3.11<br>p=0.049	*<br>q(8,100)=3.56<br>p=0.013
**21-30do**<br>(0.27±0.12)				n.s.<br>q(8,100)= 0.93<br>p=0.983	n.s.<br>q(8,100)= 1.65<br>p=0.718	n.s.<br>q(8,100)= 1.10<br>p=0.956	n.s.<br>q(8,100)=1.55<br>p=0.781	n.s.<br>q(8,100)=2.17<br>p=0.380
**35-70do**<br>(0.23±0.11)					n.s.<br>q(8,100)=0.83<br>p=0.991	n.s.<br>q(8,100)=0.25<br>p=1	n.s.<br>q(8,100)=0.72<br>p=0.996	n.s.<br>q(8,100)=1.51<br>p=0.802
**3-6mo**<br>(0.20±0.10)						n.s.<br>q(8,100)=0.51<br>p=1	n.s.<br>q(8,100)=0.09<br>p=1	n.s.<br>q(8,100)=0.83<br>p=0.991
**6-9mo**<br>(0.21±0.09)							n.s.<br>q(8,100)=0.42<br>p=1	n.s.<br>q(8,100)=1.21<br>p=0.928
**18-24mo**<br>(0.21±0.11)								n.s.<br>q(8,100)=0.89<br>p=0.987

Note: All pairwise comparisons for the 8 age groups of interest (7-10do, 13-18do, 21-30do, 35-70do, 3-6mo, 6-9mo, 18-24mo and 24-27mo) were made using Tukey's honestly significant difference (HSD) test and the obtained values of the studentized range statistic (q) were compared to critical values for n=8 means and df_{error}= 100 for a significance level of 0.05. Significance is indicated as * for p<0.05, ** for p<0.01 and *** for p<0.001. Results in each cell demonstrate level of significance followed by respective q and p values.

Table 1m

Post-hoc pairwise comparison analysis of age's significant effect on Up state CV interevent interval (i.e.i.)

**Age**<br>CV iei<br>(mean ±sd)	**7-10do**<br>(0.88±0.58)	**13-18do**<br>(0.76±0.11)	**21-30do**<br>(0.86±0.16)	**35-70do**<br>(0.86±0.10)	**3-6mo**<br>(0.89±0.22)	**6-9mo**<br>(0.78±0.18)	**18-24mo**<br>(1.01±0.25)	**24-27mo**<br>(0.84±0.24)
**7-10do**<br>(0.88±0.58)		n.s.<br>q(8,100)=0.24<br>p=1	n.s.<br>q(8,100)=1.78<br>p=0.636	n.s.<br>q(8,100)=1.81<br>p=0.614	n.s.<br>q(8,100)=1.98<br>p=0.503	n.s.<br>q(8,100)=1<br>p=0.974	*<br>q(8,100)=3.13<br>p=0.046	n.s.<br>q(8,100)=1.75<br>p=0.655
**13-18do**<br>(0.76±0.11)			n.s.<br>q(8,100)=1.78<br>p=0.637	n.s.<br>q(8,100)=1.85<br>p=0.585	n.s.<br>q(8,100)=2.06<br>p=0.448	n.s.<br>q(8,100)=0.88<br>p=0.987	*<br>q(8,100)=3.42<br>p=0.020	n.s.<br>q(8,100)=1.74<br>p=0.658
**21-30do**<br>(0.86±0.16)				n.s.<br>q(8,100)= 0.17<br>p=1	n.s.<br>q(8,100)= 0.03<br>p=1	n.s.<br>q(8,100)= 0.94<br>p=0.981	n.s.<br>q(8,100)=1.25<br>p=0.914	n.s.<br>q(8,100)=0.03<br>p=1
**35-70do**<br>(0.86±0.10)					n.s.<br>q(8,100)=0.17<br>p=1	n.s.<br>q(8,100)=0.89<br>p=0.986	n.s.<br>q(8,100)=1.65<br>p=0.721	n.s.<br>q(8,100)=0.13<br>p=1
**3-6mo**<br>(0.89±0.22)						n.s.<br>q(8,100)=1.07<br>p=0.961	n.s.<br>q(8,100)=1.52<br>p=0.793	n.s.<br>q(8,100)=0.01<br>p=1
**6-9mo**<br>(0.78±0.18)							n.s.<br>q(8,100)=2.43<br>p=0.91	n.s.<br>q(8,100)=2.41<br>p=0.985
**18-24mo**<br>(1.01±0.25)								n.s.<br>q(8,100)=1.28<br>p=0.903

Note: A one-way ANOVA (ARTool) analysis showed a significant effect of age on CV i.e.i.: $F(7,100)=2.496$, $p<0.05$. Post-hoc analysis includes all pairwise comparisons for the 8 age groups of interest (7-10do, 13-18do, 21-30do, 35-70do, 3-6mo, 6-9mo, 18-24mo and 24-27mo) using Tukey's honestly significant difference (HSD) test and the obtained values of the studentized range statistic (q) were compared to critical values for n=8 means and $df_{error}= 100$ for a significance level of 0.05. Significance is indicated as * for $p<0.05$, ** for $p<0.01$ and *** for $p<0.001$. Results in each cell demonstrate level of significance followed by respective q and p values.

Table 1n

Post-hoc pairwise comparison analysis of age's significant effect on Up state CV duration

**Age** **CV duration** (mean ±sd)	**7-10do** (0.15±0.06)	**13-18do** (0.34±0.14)	**21-30do** (0.33±0.06)	**35-70do** (0.30±0.08)	**3-6mo** (0.27±0.09)	**6-9mo** (0.25±0.09)	**18-24mo** (0.24±0.10)	**24-27mo** (0.22±0.07)
**7-10do** (0.15±0.06)		*** q(8,100)=4.58 p<0.001	*** q(8,100)=4.62 p<0.001	*** q(8,100)=4.20 p<0.01	*** q(8,100)=3.14 p=0.045	n.s. q(8,100)=2.49 p=0.211	n.s. q(8,100)=2.21 p=0.356	n.s. q(8,100)=1.60 p=0.750
**13-18do** (0.34±0.14)			n.s. q(8,100)=0.39 p=1	n.s. q(8,100)=0.64 p=0.998	n.s. q(8,100)=2.01 p=0.478	n.s. q(8,100)=2.37 p=0.269	n.s. q(8,100)=3.00 p=0.064	n.s. q(8,100)=3.07 p=0.053
**21-30do** (0.33±0.06)				n.s. q(8,100)= 0.98 p=0.976	n.s. q(8,100)= 2.23 p=0.344	n.s. q(8,100)= 2.55 p=0.187	* q(8,100)=3.13 p=0.046	* q(8,100)=3.21 p=0.037
**35-70do** (0.30±0.08)					n.s. q(8,100)=1.43 p=0.840	n.s. q(8,100)=1.85 p=0.589	n.s. q(8,100)=2.49 p=0.213	n.s. q(8,100)=2.62 p=0.163
**3-6mo** (0.27±0.09)						n.s. q(8,100)=0.56 p=0.999	n.s. q(8,100)=1.12 p=0.950	n.s. q(8,100)=1.44 p=0.835
**6-9mo** (0.25±0.09)							n.s. q(8,100)=0.47 p=1	n.s. q(8,100)=0.86 p=0.989
**18-24mo** (0.24±0.10)								n.s. q(8,100)=0.47 p=1

Note: A one-way ANOVA (ARTool) analysis showed a significant effect of age on CV duration.: $F(7,100)=5.510$, $p<0.001$. Posthoc analysis includes all pairwise comparisons for the 8 age groups of interest (7-10do, 13-18do, 21-30do, 35-70do, 3-6mo, 6-9mo, 18-24mo and 24-27mo) using Tukey's honestly significant difference (HSD) test and the obtained values of the studentized range statistic (q) were compared to critical values for n=8 means and df_{error}= 100 for a significance level of 0.05. Significance is indicated as * for p<0.05, ** for p<0.01 and *** for p<0.001. Results in each cell demonstrate level of significance followed by respective q and p values.

Table 1o

Post-hoc pairwise comparison analysis of age's significant effect on Up state CV amplitude (ANOVA (ARTool), F(7,100)=7.808, p<0.001)

**Age**<br>CV amplitude<br>(mean ±sd)	**7-10do**<br>(0.03±0.03)	**13-18do**<br>(0.31±0.05)	**21-30do**<br>(0.28±0.04)	**35-70do**<br>(0.28±0.07)	**3-6mo**<br>(0.22±0.03)	**6-9mo**<br>(0.32±0.13)	**18-24mo**<br>(0.27±0.07)	**24-27mo**<br>(0.26±0.08)
**7-10do**<br>(0.03±0.03)		***<br>q(8,100)=6<br>p<0.001	***<br>q(8,100)=4.97<br>p<0.001	***<br>q(8,100)=5.12<br>p<0.01	n.s.<br>q(8,100)=3.00<br>p=0.065	***<br>q(8,100)=5.74<br>p<0.01	***<br>q(8,100)=4.78<br>p<0.01	**<br>q(8,100)=4.42<br>p=0.01
**13-18do**<br>(0.31±0.05)			n.s.<br>q(8,100)=0.73<br>p=0.996	n.s.<br>q(8,100)=1.29<br>p=0.902	**<br>q(8,100)=3.97<br>p=0.003	n.s.<br>q(8,100)=0.22<br>p=1	n.s.<br>q(8,100)=1.69<br>p=0.694	n.s.<br>q(8,100)=1.36<br>p=0.873
**21-30do**<br>(0.28±0.04)				n.s.<br>q(8,100)= 0.41<br>p=1	n.s.<br>q(8,100)= 2.80<br>p=0.106	n.s.<br>q(8,100)= 0.52<br>p=1	n.s.<br>q(8,100)=0.77<br>p=0.994	n.s.<br>q(8,100)=0.58<br>p=0.999
**35-70do**<br>(0.28±0.07)					n.s.<br>q(8,100)=2.79<br>p=0.108	n.s.<br>q(8,100)=1.03<br>p=0.968	n.s.<br>q(8,100)=0.42<br>p=1	n.s.<br>q(8,100)=0.25<br>p=1
**3-6mo**<br>(0.22±0.03)						n.s.<br>q(8,100)=3.65<br>p=0.010	n.s.<br>q(8,100)=2.36<br>p=0.274	n.s.<br>q(8,100)=2.13<br>p=0.400
**6-9mo**<br>(0.32±0.13)							n.s.<br>q(8,100)=1.43<br>p=0.842	n.s.<br>q(8,100)=1.14<br>p=0.946
**18-24mo**<br>(0.27±0.07)								n.s.<br>q(8,100)=0.12<br>p=1

Note: A one-way ANOVA (ARTool) analysis showed a significant effect of age on CV amplitude (F(7,100)=7.808, p<0.001). Posthoc analysis includes all pairwise comparisons for the 8 age groups of interest (7-10do, 13-18do, 21-30do, 35-70do, 3-6mo, 6-9mo, 18-24mo and 24-27mo) using Tukey's honestly significant difference (HSD) test and the obtained values of the studentized range statistic (q) were compared to critical values for n=8 means and df_{error}= 100 for a significance level of 0.05. Significance is indicated as * for p<0.05, ** for p<0.01 and *** for p<0.001. Results in each cell demonstrate level of significance followed by respective q and p values.

Table 1p

Post-hoc pairwise comparison analysis of age's significant effect on Up state CV rectified area

Age<br>CV rectified area<br>(mean ±sd)	7-10do<br>(0.12±0.04)	13-18do<br>(0.35±0.11)	21-30do<br>(0.35±0.06)	35-70do<br>(0.33±0.11)	3-6mo<br>(0.30±0.10)	6-9mo<br>(0.31±0.12)	18-24mo<br>(0.29±0.12)	24-27mo<br>(0.23±0.05)
**7-10do**<br>(0.12±0.04)		***<br>q(8,100)=5.31<br>p<0.001	***<br>q(8,100)=4.84<br>p<0.001	***<br>q(8,100)=5.03<br>p<0.01	**<br>q(8,100)=3.96<br>p=0.003	**<br>q(8,100)=4.15<br>p=0.002	**<br>q(8,100)=3.94<br>p=0.004	n.s.<br>q(8,100)=2.20<br>p=0.363
**13-18do**<br>(0.35±0.11)			n.s.<br>q(8,100)=0.13<br>p=1	n.s.<br>q(8,100)=0.54<br>p=0.999	n.s.<br>q(8,100)=1.94<br>p=0.527	n.s.<br>q(8,100)=1.27<br>p=0.909	n.s.<br>q(8,100)=1.84<br>p=0.598	*<br>q(8,100)=3.16<br>p=0.041
**21-30do**<br>(0.35±0.06)				n.s.<br>q(8,100)= 0.35<br>p=1	n.s.<br>q(8,100)= 1.61<br>p=0.744	n.s.<br>q(8,100)= 1.03<br>p=0.969	n.s.<br>q(8,100)=1.52<br>p=0.793	n.s.<br>q(8,100)=2.81<br>p=0.105
**35-70do**<br>(0.33±0.11)					n.s.<br>q(8,100)=1.46<br>p=0.826	n.s.<br>q(8,100)=0.79<br>p=0.993	n.s.<br>q(8,100)=1.36<br>p=0.872	n.s.<br>q(8,100)=2.80<br>p=0.107
**3-6mo**<br>(0.30±0.10)						n.s.<br>q(8,100)=0.54<br>p=0.999	n.s.<br>q(8,100)=0.06<br>p=1	n.s.<br>q(8,100)=1.61<br>p=0.746
**6-9mo**<br>(0.31±0.12)							n.s.<br>q(8,100)=0.47<br>p=1	n.s.<br>q(8,100)=1.96<br>p=0.517
**18-24mo**<br>(0.29±0.12)								n.s.<br>q(8,100)=1.63<br>p=0.734

Note: A one-way ANOVA (ARTool) analysis showed a significant effect of age on CV rectified area (ANOVA (ARTool), $F(7,100)=5.818$, $p<0.001$). Posthoc analysis includes all pairwise comparisons for the 8 age groups of interest (7-10do, 13-18do, 21-30do, 35-70do, 3-6mo, 6-9mo, 18-24mo and 24-27mo) using Tukey's honestly significant difference (HSD) test and the obtained values of the studentized range statistic (q) were compared to critical values for n=8 means and df_{error}= 100 for a significance level of 0.05. Significance is indicated as * for $p<0.05$, ** for $p<0.01$ and *** for $p<0.001$. Results in each cell demonstrate level of significance followed by respective q and p values.

Table 1q

Post-hoc pairwise comparison analysis of age's significant effect on Up state CV normalized theta

Age<br>CV theta<br>(mean ±sd)	**7-10do**<br>(0.23±0.15)	**13-18do**<br>(0.43±0.07)	**21-30do**<br>(0.40±0.12)	**35-70do**<br>(0.47±0.08)	**3-6mo**<br>(0.44±0.13)	**6-9mo**<br>(0.46±0.11)	**18-24mo**<br>(0.48±0.11)	**24-27mo**<br>(0.47±0.11)
**7-10do**<br>(0.23±0.15)		n.s.<br>q(8,100)=2.56<br>p=0.182	n.s.<br>q(8,100)=2.15<br>p=0.389	**<br>q(8,100)=3.77<br>p=0.06	n.s.<br>q(8,100)=2.81<br>p=0.104	*<br>q(8,100)=3.49<br>p=0.016	**<br>q(8,100)=4.05<br>p=0.003	n.s.<br>q(8,100)=2.92<br>p=0.080
**13-18do**<br>(0.43±0.07)			n.s.<br>q(8,100)=0.28<br>p=1	n.s.<br>q(8,100)=1.33<br>p=0.884	n.s.<br>q(8,100)=0.14<br>p=1	n.s.<br>q(8,100)=1.12<br>p=0.950	n.s.<br>q(8,100)=1.66<br>p=0.712	n.s.<br>q(8,100)=0.60<br>p=0.999
**21-30do**<br>(0.40±0.12)				n.s.<br>q(8,100)= 1.50<br>p=0.808	n.s.<br>q(8,100)= 0.42<br>p=1	n.s.<br>q(8,100)= 1.30<br>p=0.896	n.s.<br>q(8,100)=1.79<br>p=0.628	n.s.<br>q(8,100)=0.81<br>p=0.992
**35-70do**<br>(0.47±0.08)					n.s.<br>q(8,100)=1.30<br>p=0.898	n.s.<br>q(8,100)=0.13<br>p=1	n.s.<br>q(8,100)=0.34<br>p=0.872	n.s.<br>q(8,100)=0.59<br>p=0.999
**3-6mo**<br>(0.44±0.13)						n.s.<br>q(8,100)=1.07<br>p=0.962	n.s.<br>q(8,100)=1.65<br>p=0.718	n.s.<br>q(8,100)=0.51<br>p=1
**6-9mo**<br>(0.46±0.11)							n.s.<br>q(8,100)=0.45<br>p=1	n.s.<br>q(8,100)=0.44<br>p=1
**18-24mo**<br>(0.48±0.11)								n.s.<br>q(8,100)=0.88<br>p=0.987

Note: A one-way ANOVA (ARTool) analysis showed a significant effect of age on CV normalized theta power ($F(7,100)=2.981$, $p<0.01$). Posthoc analysis included all pairwise comparisons for the 8 age groups of interest (7-10do, 13-18do, 21-30do, 35-70do, 3-6mo, 6-9mo, 18-24mo and 24-27mo) using Tukey's honestly significant difference (HSD) test and the obtained values of the studentized range statistic (q) were compared to critical values for n=8 means and df_{error}= 100 for a significance level of 0.05. Significance is indicated as * for $p<0.05$, ** for $p<0.01$ and *** for $p<0.001$. Results in each cell demonstrate level of significance followed by respective q and p values.

Table 1r

Post-hoc pairwise comparison analysis of age's significant effect on Up state CV normalized beta power (ANOVA), F(7,100)=2.430, p<0.05)

Age<br>CV beta<br>(mean ±sd)	**7-10do**<br>(0.37±0.13)	**13-18do**<br>(0.35±0.05)	**21-30do**<br>(0.37±0.07)	**35-70do**<br>(0.42±0.09)	**3-6mo**<br>(0.45±0.08)	**6-9mo**<br>(0.43±0.09)	**18-24mo**<br>(0.45±0.09)	**24-27mo**<br>(0.46±0.14)
**7-10do**<br>(0.37±0.13)		n.s.<br>q(8,100)=0.32<br>p=1	n.s.<br>q(8,100)=0.08<br>p=1	n.s.<br>q(8,100)=1.32<br>p=0.889	n.s.<br>q(8,100)=2.08<br>p=0.433	n.s.<br>q(8,100)=1.35<br>p=0.878	n.s.<br>q(8,100)=2.01<br>p=0.481	n.s.<br>q(8,100)=2.01<br>p=0.480
**13-18do**<br>(0.35±0.05)			n.s.<br>q(8,100)=0.43<br>p=1	n.s.<br>q(8,100)=1.96<br>p=0.516	n.s.<br>q(8,100)=2.89<br>p=0.085	n.s.<br>q(8,100)=1.94<br>p=0.529	n.s.<br>q(8,100)=2.78<br>p=0.113	n.s.<br>q(8,100)=2.64<br>p=0.153
**21-30do**<br>(0.37±0.07)				n.s.<br>q(8,100)= 1.32<br>p=0.889	n.s.<br>q(8,100)= 2.15<br>p=0.393	n.s.<br>q(8,100)= 1.35<br>p=0.878	n.s.<br>q(8,100)=2.06<br>p=0.446	n.s.<br>q(8,100)=2.04<br>p=0.458
**35-70do**<br>(0.42±0.09)					n.s.<br>q(8,100)=0.94<br>p=0.982	n.s.<br>q(8,100)=0.11<br>p=1	n.s.<br>q(8,100)=0.86<br>p=0.989	n.s.<br>q(8,100)=0.97<br>p=0.977
**3-6mo**<br>(0.45±0.08)						n.s.<br>q(8,100)=0.76<br>p=0.995	n.s.<br>q(8,100)=0.05<br>p=1	n.s.<br>q(8,100)=0.19<br>p=1
**6-9mo**<br>(0.43±0.09)							n.s.<br>q(8,100)=0.69<br>p=0.997	n.s.<br>q(8,100)=0.83<br>p=0.991
**18-24mo**<br>(0.45±0.09)								n.s.<br>q(8,100)=0.23<br>p=1

Note: A one-way ANOVA analysis showed a significant effect of age on CV normalized beta power F(7,100)=2.981, p<0.01). Posthoc analysis includes all pairwise comparisons for the 8 age groups of interest (7-10do, 13-18do, 21-30do, 35-70do, 3-6mo, 6-9mo, 18-24mo and 24-27mo) using Tukey's honestly significant difference (HSD) test and the obtained values of the studentized range statistic (q) were compared to critical values for n=8 means and df_{error}= 100 for a significance level of 0.05. Significance is indicated as * for p<0.05, ** for p<0.01and *** for p<0.001. Results in each cell demonstrate level of significance followed by respective q and p values.

Table 1s

Post-hoc pairwise comparison analysis of age's significant effect on Up state CV normalized gamma power

Age<br>CV gamma<br>(mean ±sd)	7-10do<br>(0.37±0.13)	13-18do<br>(0.35±0.05)	21-30do<br>(0.37±0.07)	35-70do<br>(0.42±0.09)	3-6mo<br>(0.45±0.08)	6-9mo<br>(0.43±0.09)	18-24mo<br>(0.45±0.09)	24-27mo<br>(0.46±0.14)
**7-10do**<br>(0.37±0.13)		n.s.<br>q(8,100)=1.48<br>p=0.814	n.s.<br>q(8,100)=2.46<br>p=0.226	*<br>q(8,100)=3.15<br>p=0.043	n.s.<br>q(8,100)=2.34<br>p=0.281	**<br>q(8,100)=3.77<br>p=0.007	**<br>q(8,100)=4.41<br>p=0.001	**<br>q(8,100)=3.67<br>p=0.009
**13-18do**<br>(0.35±0.05)			n.s.<br>q(8,100)=1.23<br>p=0.922	n.s.<br>q(8,100)=1.92<br>p=0.540	n.s.<br>q(8,100)=0.94<br>p=0.982	n.s.<br>q(8,100)=2.69<br>p=0.139	*<br>q(8,100)=3.42<br>p=0.020	n.s.<br>q(8,100)=2.61<br>p=0.163
**21-30do**<br>(0.37±0.07)				n.s.<br>q(8,100)= 0.47<br>p=1	n.s.<br>q(8,100)= 0.45<br>p=1	n.s.<br>q(8,100)= 1.25<br>p=0.914	n.s.<br>q(8,100)=1.82<br>p=0.609	n.s.<br>q(8,100)=1.28<br>p=0.903
**35-70do**<br>(0.42±0.09)					n.s.<br>q(8,100)=1.09<br>p=0.958	n.s.<br>q(8,100)=0.93<br>p=0.983	n.s.<br>q(8,100)=1.57<br>p=0.766	n.s.<br>q(8,100)=0.98<br>p=0.977
**3-6mo**<br>(0.45±0.08)						n.s.<br>q(8,100)=1.96<br>p=0.516	n.s.<br>q(8,100)=2.70<br>p=0.134	n.s.<br>q(8,100)=1.93<br>p=0.537
**6-9mo**<br>(0.43±0.09)							n.s.<br>q(8,100)=0.54<br>p=0.999	n.s.<br>q(8,100)=0.11<br>p=1
**18-24mo**<br>(0.45±0.09)								n.s.<br>q(8,100)=0.38<br>p=1

Note: A one-way ANOVA analysis showed a significant effect of age on CV normalized gamma power $F(7,100)=4.410$, $p<0.001$). Posthoc analysis includes all pairwise comparisons for the 8 age groups of interest (7-10do, 13-18do, 21-30do, 35-70do, 3-6mo, 6-9mo, 18-24mo and 24-27mo) using Tukey's honestly significant difference (HSD) test and the obtained values of the studentized range statistic (q) were compared to critical values for n=8 means and df_{error}= 100 for a significance level of 0.05. Significance is indicated as * for $p<0.05$, ** for $p<0.01$ and *** for $p<0.001$. Results in each cell demonstrate level of significance followed by respective q and p values.
