# Supplementary material for: Spontaneous Up states in vitro: a single-metric index of the functional maturation and regional differentiation of the cerebral cortex
Source: Front Neural Circuits. 2015 Oct 13;9:59. doi: 10.3389/fncir.2015.00059 (PMC4603250; doi:10.3389/fncir.2015.00059)
Supplement: Supplementary file 8 [file Table2.PDF]

Table 2: Comparing Up states in the S1BF and M1 cortex.

Measurement	Cortex S1BF (mean±SD)	Cortex M1 (mean±SD)	Significance
**Occurrence (min^{-1})**	0.87±0.61	0.49±0.32	* [U(18,22))=124, p=0.044]
**Duration (sec)**	1.25±0.20	1.48±0.43	* [t(23)=-2.082 p=0.049]
**% Time in Up state**	1.80±1.23%	1.24±0.90	* [χ^2(1)= 6.12 p=0.013]
**Amplitude (µV)**	-54±24	-49±16	n.s. [U(18,22)=187.5, p=0.775]
**Rectified Area (µV^2)**	0.13±0.05	0.10±0.05	n.s. [t(38)=-0.768, p=0.447]
**Up state Index**	0.09±0.05	0.05±0.03	** [t(38)=3.318, p=0.002]
**Normalized delta**	0.55±0.13	0.49±0.14	n.s. [t(38)=1.457, p=0.153]
**Normalized theta**	0.17±0.04	0.17±0.03	n.s. [t(38)=0.311, p=0.758]
**Normalized alpha**	0.07±0.03	0.07±0.03	n.s. [U(18,22))=166, p=0.384]
**Normalized beta**	0.11±0.04	0.14±0.07	* [t(38)=-2.051, p=0.047]
**Normalized gamma**	0.10±0.07	0.13±0.06	* [U(18,22))=116, p=0.026]
**Normalized lower frequencies**	0.72±0.11	0.66±0.13	n.s. [t(38)=1.733, p=0.091]
**Normalized higher frequencies**	0.21±0.10	0.27±0.12	* [U(18,22))=113, p=0.021]

Comparisons were performed for two independent samples using either Student's t-test if data was normally distributed or the equivalent non-parametric test (Mann Whitney) if the data were not. Significance is indicated as non significant (n.s.) for p >0.05, * for p<0.05, ** for p<0.01 and *** for p<0.001. Results in middle columns compare mean±SD (standard deviation) of S1 vs M1 cortex, while in the right end column each cell demonstrates level of significance followed either by respective t and p values for df=38 (except if adjusted when variances are unequal) with Student's t-test or W and p values for df=18 and 22 with the Mann-Whitney test.
